# Global Metabolomic Profiling of Acute Myocarditis Caused by *Trypanosoma cruzi* Infection

**DOI:** 10.1371/journal.pntd.0003337

**Published:** 2014-11-20

**Authors:** Núria Gironès, Sofía Carbajosa, Néstor A. Guerrero, Cristina Poveda, Carlos Chillón-Marinas, Manuel Fresno

**Affiliations:** 1 Centro de Biología Molecular Severo Ochoa, CSIC-UAM, Madrid, Spain; 2 Instituto de Investigación Sanitaria de la Princesa, Madrid, Spain; Albert Einstein College of Medicine, United States of America

## Abstract

Chagas disease is caused by *Trypanosoma cruzi* infection, being cardiomyopathy the more frequent manifestation. New chemotherapeutic drugs are needed but there are no good biomarkers for monitoring treatment efficacy. There is growing evidence linking immune response and metabolism in inflammatory processes and specifically in Chagas disease. Thus, some metabolites are able to enhance and/or inhibit the immune response. Metabolite levels found in the host during an ongoing infection could provide valuable information on the pathogenesis and/or identify deregulated metabolic pathway that can be potential candidates for treatment and being potential specific biomarkers of the disease. To gain more insight into those aspects in Chagas disease, we performed an unprecedented metabolomic analysis in heart and plasma of mice infected with *T. cruzi*. Many metabolic pathways were profoundly affected by *T. cruzi* infection, such as glucose uptake, sorbitol pathway, fatty acid and phospholipid synthesis that were increased in heart tissue but decreased in plasma. Tricarboxylic acid cycle was decreased in heart tissue and plasma whereas reactive oxygen species production and uric acid formation were also deeply increased in infected hearts suggesting a stressful condition in the heart. While specific metabolites allantoin, kynurenine and p-cresol sulfate, resulting from nucleotide, tryptophan and phenylalanine/tyrosine metabolism, respectively, were increased in heart tissue and also in plasma. These results provide new valuable information on the pathogenesis of acute Chagas disease, unravel several new metabolic pathways susceptible of clinical management and identify metabolites useful as potential specific biomarkers for monitoring treatment and clinical severity in patients.

## Author Summary

Chagas disease, caused by *Trypanosoma cruzi*, is the most common cause of cardiomyopathy in Latin America. After the acute phase myocarditis, the disease becomes asymptomatic, and many years later some patients may develop Chagas cardiomyopathy. Treatment is available, but causes side effects. Thus, new drugs are needed, but there are no good tools to assess treatment efficacy. We performed the first global metabolomics analysis in heart tissue and plasma from mice infected with *T. cruzi* along the infection. We have identified more than 200 biochemicals (around 2/3 of total) that significantly differed in heart and 100 (around 1/3) in plasma. Some of those show extremely marked and highly significant differences. More importantly, our results unravel many new aspects of metabolic alterations that can be useful for: a) better understanding the pathogenesis of this disease; b) provide new clinic-pathological data of *T. cruzi* infection that can be potentially used for a better clinical management of Chagasic patients; c) define several metabolites as potential biomarkers of this disease. Thus, our study identifies common biomarkers of cardiomyopathy but more importantly specific candidate biomarkers of acute Chagas Disease Cardiomyopathy, not observed in a clinically similar disease Idiopathic Dilated Cardiomiopathy. Allantoin, kynurenine and p-cresol sulfate, that can be easily detected in serum, are highly promising candidates. In summary, we think our results would help to better understand the pathogenesis and management of the disease and offer new candidate biomarkers for monitoring the severity of the disease and treatment efficacy.

## Introduction

Chagas disease, caused by the protozoan parasite *Trypanosoma cruzi*, affects approximately 8 million people worldwide [1] and kills more than 15,000 each year, thus representing a major cause of morbidity and mortality in endemic countries [2]. Chagasic cardiomyopathy is the most serious and frequent manifestation in *T. cruzi* infected patients in the chronic phase of the disease. Acute Chagas disease is an often nonspecific and frequently unrecognized condition, but acute infection associated to congenital cases and oral transmission can have a fatal outcome in humans [3]. Myocarditis, although uncommonly reported and difficult to diagnose, is uniformly present during acute infection. Moreover, endomyocardial biopsies taken when patients are diagnosed during this phase of the disease consistently reveal acute myocarditis, even if the patient is asymptomatic [4]. The ranges of acute cardiac pathogenesis are characterized by pericardial effusion, pericarditis, ventricular enlargement with dysfunction or congestive heart failure or both [5]. In the chronic phase, myocardial inflammation associated to mononuclear infiltrate is a common finding in histological sections, although the spatial association between parasites and inflammatory infiltrate is controversial and manifests as heart failure, arrhythmia, heart block, thromboembolism, stroke, and sudden death. Chronic Chagasic Cardiomyopathy is characterized by its severity, as well as by a worse prognosis when compared with other cardiomyopathies [6].

*T. cruzi* has a complex life cycle involving several stages in both vertebrates and insect vectors. It infects and replicates in macrophages and cardiomyocytes as well as many other cell types [2]. However, the pathogenesis is thought to be dependent on an immune-inflammatory reaction to a low-grade infection [7], [8]. To date no vaccine is available [9] and current drugs have many side effects [10]. Some chronic asymptomatic patients are currently treated with those drugs, although its efficacy has not been clearly demonstrated yet, basically because there is no useful surrogate marker for clinical improvement and therapeutic efficacy. In the meantime, follow up of the treated patients by PCR turned out to be the only way to guide physicians to deal with the disease [11], [12]. Several studies on biomarkers of cure have been proposed based on seronegativization of antibodies, lytic antibodies, hemostatic parameters, natriuretic peptides, recombinant antigens and proteomic assays [13] but none of them seems to be particularly sensitive. Thus, new biomarkers are needed to monitor responses to treatment.

Global metabolomic analysis is a powerful tool to understand the pathophysiology of parasite infection as well as to identify biomarkers of disease, as it has been done for dilated cardiomyopathy [14]. Thus, identification of metabolic conditions could provide a deeper knowledge of the pathophysiology and the immunopathology as well of being of clinical value, since restoration of those metabolites to basal levels could be beneficial for the host during acute infection. On the other hand, they could be also useful as biomarkers. The current paucity of effective preventive or therapeutic options is another strong motivation for the study of host-parasite metabolism interplay.

Moreover, metabolic function can clearly affect the activity of the immune system [15], [16], [17]. In this regard, in acute *T. cruzi* infection there are major immunological changes [18], that in some cases are linked to the production of amino acid metabolites, as kynurenine, a product of indolamine 2,3 dioxygenase (IDO) enzymatic activity from tryptophan, which is supposed to control parasite growth [19], [20]. In addition, the levels of L-arginine, modulated by Arginase enzymatic activity, deeply affect the activity of T lymphocytes in experimental *T. cruzi* infection in mice [21]. Thus, the knowledge of the metabolic alterations will help to understand the course of the infection.

Since *T. cruzi* is able to replicate intracellularly, it will likely affect infected host cell metabolism. Thus, metabolic changes were observed *in vitro* after infection with the parasite in endothelial cells [22]. In addition, Garg et al performed microarray analysis of the cardiomyocyte mitochondrial metabolism during *in vivo* infection showing deficiencies in mitochondrial oxidative phosphorylation-mediated ATP generation that plays an important role in cardiac homeostasis [23]. More recently, Caradonna et al. using a genome-scale functional screen identified interconnected metabolic networks centered around host energy production, nucleotide metabolism, pteridine biosynthesis, and fatty acid oxidation as key processes that fuel intracellular *T. cruzi* growth in HeLa cells *in vitro* [24].

However, there are no reports to date on global metabolomic analysis during *in vivo T. cruzi* infection. Moreover, metabolite levels during infection may reflect not only host metabolism but also the possible contribution of parasite-derived metabolites, which has never been assessed before. Thus, here we studied the metabolic profile in heart and plasma from *T. cruzi* infected mice. The results unravel many new aspects of metabolic alterations that can be useful for better understanding the pathogenesis of this disease and to better control *T. cruzi* infection as well as showing the potential of particular metabolites as biomarkers of this disease.

## Materials and Methods

### Mice and parasites

Young adult (6 to 8-week-old) BALB/c female mice were transported from Charles River Laboratories and hosted in a controlled environment. *In vivo* infections performed with Y *T. cruzi* strain (obtained from Dr. John David, Department of medicine, Harvard Medical School, Boston, Massachusetts, U.S.A.). Blood trypomastigotes were routinely maintained by infecting IFNγ receptor-deficient mice (129 Ifngrttm1Agt/J) [25] purifying them from their blood. These mice were a gift from Manfred Kopf (Max-Planck-Institute for Immunobiology, Freiburg). Parasitemia levels were checked every two-three days by microscopic inspection and counting of parasites in a 5 µl drop of the tail vein blood as described [26]. Real time qPCR was performed as described [27].

### Ethics statement

This study was carried out in strict accordance with the European Commission legislation for the protection of animals used for scientific purposes (Directive 2010/63/EU). Mice were maintained under pathogen-free conditions at the Centro de Biología Molecular Severo Ochoa (CSIC-UAM) animal facility. The protocol for the treatment of the animals was approved by the “Comité de Ética de Investigación de la Universidad Autónoma de Madrid”, Spain (permit CEI-47-899). Animals had unlimited access to food and water. They were euthanized in a CO_2_ chamber and all efforts were made to minimize their suffering.

### Experimental design

Global biochemical profiles were determined in methanol extracts derived from mouse heart tissue and plasma from two independent experiments including uninfected mice (n = 6) and mice infected with 2,000 blood trypomastigotes of the Y strain of *T. cruzi* (see supplemental experimental information for details), and sacrificed at 14 (n = 6) and 21 days post-infection (n = 6). Plasma and heart tissue were elicited. To prevent blood coagulation, syringes were impregnated with heparin, and plasma was obtained from the supernatant immediately after centrifugation of the blood and frozen at −80°C. Hearts were perfused with 10 ml of phosphate buffered saline (PBS) and heparin to remove blood and immediately frozen at −80°C. Samples were prepared following instructions from Metabolon (see supplemental experimental information for details).

### Parasite DNA quantitative real time PCR

At different days post infection mice were euthanized in a CO_2_ chamber and blood and heart tissue were collected. Hearts were perfused with PBS and heparin (1 U/ml) and were minced into small pieces with a sterile scalpel and DNA was isolated with High PurePCR Template preparation Kit, Roche. For *T. cruzi* detection, we followed the qPCR assay described by [27]. Briefly, 100, 10, 1, 0.1 and 0.01 pg of DNA purified from Y strain epimastigotes were used to generate the standard curve. Experimental heart tissue qPCR reactions contained 100 ng of genomic DNA. Murine *Tnf* gene qPCR reactions were set up for normalization and expressed as pg Parasite DNA/ng heart tissue DNA.

### Biochemical sample preparation

Samples were prepared following Metabolon's instructions. Briefly, heart tissue was defrosted at room temperature (RT) and cut with sterile surgical blade. Approximately 80 mg of tissue were weighed and disposed in 2 ml eppendorf tubes. Heart tissue was heat-inactivated at 50°C for 30 min. 1600 µl of the extraction methanol solvent A (containing standards resuspended in 80% HPLC grade Methanol; Sigma Aldrich 494291, CAS 67-56-1) were added to the sample. Plasma was defrosted at RT. 100 µl of plasma was transferred to a new tube and any parasites present in plasma were heat-inactivated at 50°C for 30 min. Then, plasma was combined with 450 ul of extraction Metabolon solvent B (containing standards resuspended in 100% HPLC grade Methanol; Sigma Aldrich 494291, CAS 67-56-1). After incubation for 24 h at RT, samples were stored at −80°C until shipment in dry ice.

### Metabolomic analysis

The extracted samples were split into equal parts for analysis on the gas chromatography (GC)/mass spectrometry (MS) and liquid chromatography (LC)/MS platforms. Instrument variability was determined by calculating the median relative standard deviation (RSD) for the internal standards that were added to each sample prior to injection into the mass spectrometers. Overall process variability was determined by calculating the median RSD for all endogenous metabolites (i.e., non-instrument standards) present in 100% of the Matrix samples, which are technical replicates of pooled samples. Values were normalized in terms of raw area counts (OrigScale). For a single day run, this is equivalent to the raw data. Each biochemical in OrigScale is rescaled to set the median equal to 1 and expressed as imputed normalized counts for each biochemical (ScaledImpData).

### Principal components analysis

Principal component analysis (PCA) is a mathematical procedure that uses an orthogonal transformation to convert a set of observations of possibly correlated variables into a set of values of linearly uncorrelated variables called principle components. This transformation is defined in such a way that the first principal component has the largest possible variance (that is, accounts for as much of the variability in the data as possible), and the second component in turn has the highest variance possible under the constraint that it is orthogonal to (i.e., uncorrelated with) the preceding component. Thus, data set interpretation is that the stratification of metabolites by component 1 may have the greatest contribution to separating the metabolic signature of these samples followed by component 2.

### Statistical analysis

Pair-wise comparisons of data from infected mice respect non-infected (0 dpi) were performed by Welch's two sample t-tests using the program “R” http://cran.r-project.org/.

## Results

### Experimental *T. cruzi* infection

**Figure 1:**
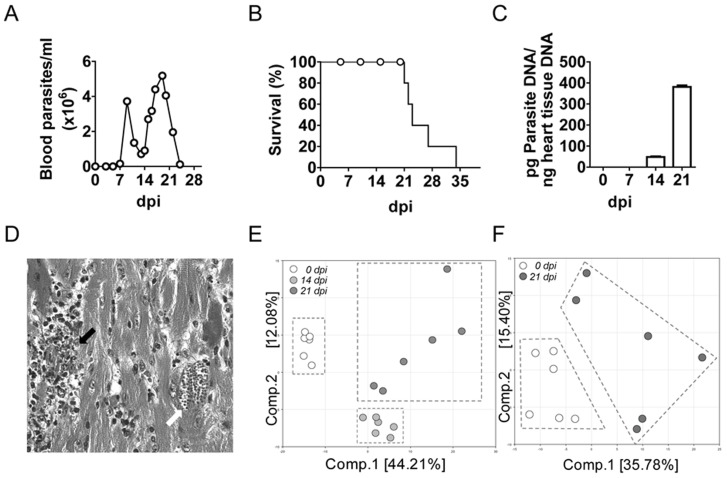
Parasite burden and mice survival during *T. cruzi* infection. Parasite burden in blood and survival were monitored every two-three days as described in material and methods section. (A) Parasitemia. (B) Parasite DNA quantification by qPCR as described in the methods. (C) Mice survival. (D) Representative heart tissue section stained with H&E showing amastigote nests (white arrow) and mononuclear cell infiltration (black arrow). (E) Principal component analysis in heart tissue samples. (F) Principal component analysis in plasma samples.

Infection of BALB/c mice with the Y strain of *T. cruzi* produced high parasitemia in the second and third week post-infection (Figure 1A) and no survival was observed by 34 dpi (Figure 1B). In a simultaneous experiment parasite DNA in heart tissue increased up to 21 days post-infection (dpi), the last day analyzed before the end of the experiment (Figure 1C). Besides, histological analysis of the heart at 21 dpi showed heart damage, with intense myocarditis and amastigote nests (Figure 1D).

### Metabolic changes

We selected samples at 14 dpi and 21 dpi from heart tissue and plasma 21 dpi for metabolic analysis, corresponding to the acute phase of infection. We performed a global metabolomic analysis and we detected a total of 325 compounds of known identity (named biochemicals) in heart extract and 306 compounds in plasma extract (Table S1). Following log transformation and imputation with minimum observed values for each compound, Welch's two-sample *t*-test was used to identify biochemicals that differed significantly between experimental groups. Our analysis identified more than 200 biochemicals (around 2/3) that differed significantly in heart and 100 (around 1/3) in plasma.

Multiple metabolites were altered upon *T. cruzi* infection, and Principal component analysis (PCA) revealed that, in heart tissue, *T. cruzi* infection was accompanied by distinct biochemical changes compared to control animals (Figure 1E). Components 1 and 2 could be decomposed into several metabolite levels that explained 44.21% and 12.08% of the total variation, respectively. Thus, samples from control and infected mice at 14 dpi grouped separately in the analysis, although more heterogeneity was observed by day 21 post-infection in heart tissue (Figure 1E). Similarly, in plasma, components 1 and 2 explained 35.71% and 15.40%, respectively, and discriminated biochemicals in control and infected mice at 21 dpi, despite the greater heterogeneity observed in this case between individual mice (Figure 1F). PCA components used for the heart and plasma are shown in supplementary tables S2 and S3, respectively, which list the metabolites contributing to the stratification of the PCA profiles. In this files, the coefficient value of each metabolite for component 1 and component 2 are represented. The larger the positive or negative coefficients value for a given metabolite, the greater its contribution to separating the metabolic profiles and thus the better candidate it may be as a biomarker. We will highlight below the more relevant changes in several pathways.

### Glucose metabolism

**Figure 2:**
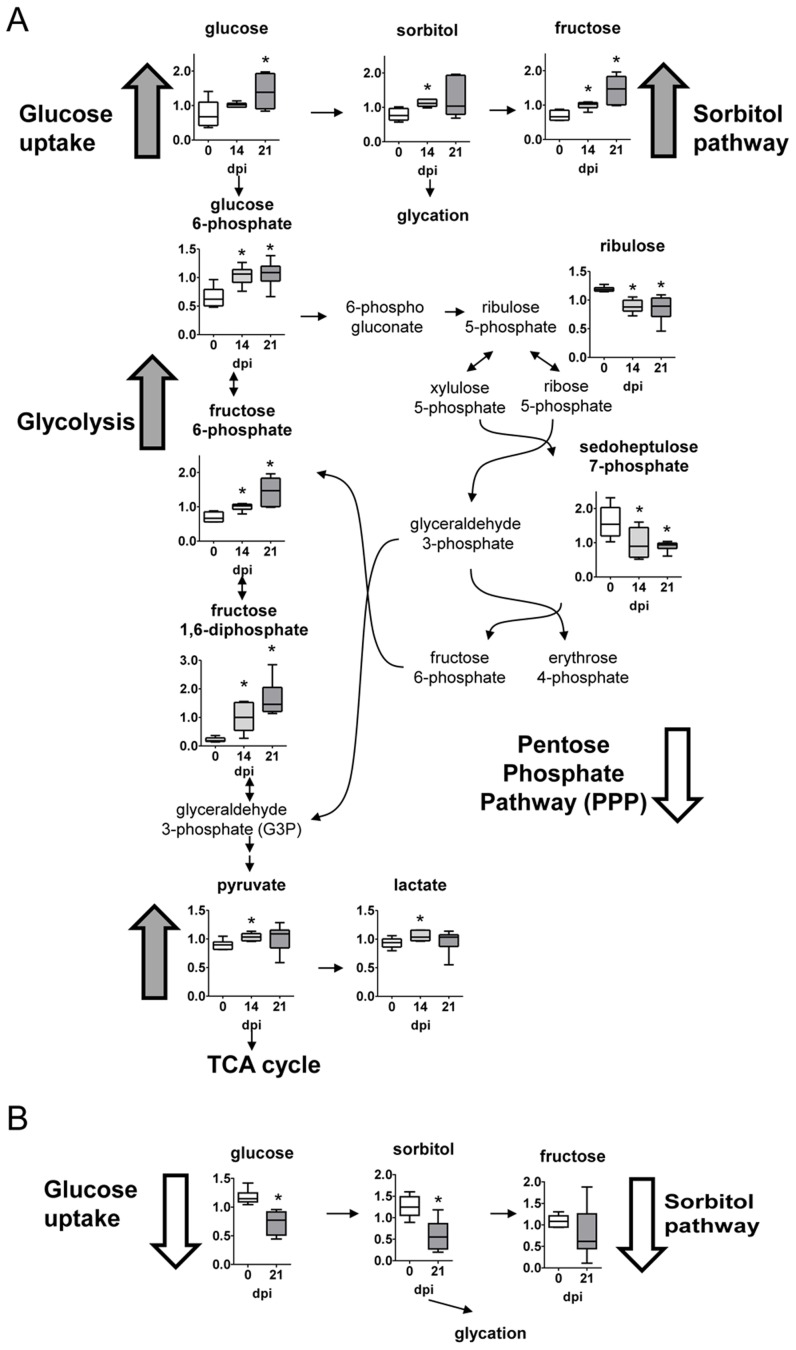
Carbohydrate pathways. (A) Graphs represent the ScaledImpData of different biochemicals of the glycolytic, sorbitol and pentose phosphate pathways from heart tissue. (B) Same as in A from plasma samples. Samples from control uninfected mice are in white boxes, from mice sacrificed at 14 dpi in light gray boxes and from mice sacrificed at 21 dpi in dark grey boxes. Statistically significant differences respect to uninfected mouse samples are denoted, *p≤0.05.

Compared to uninfected counterparts, glucose levels were elevated in heart tissue by days 14 and 21 post-infection (Figure 2A). This observation likely reflects an increase in glucose uptake considering glucose levels declined in infected plasma over time (Figure 2B). This also agrees with the observed increase of the sorbitol pathway metabolites, sorbitol and fructose, in the heart. Besides, multiple glycolytic intermediates including glucose-6 phosphate and fructose-6-phosphate and the end products pyruvate and lactate were also significantly elevated in infected heart tissue, but not in plasma (Figure 2A and B, respectively). In contrast, reduced glucose shuttling to the pentose phosphate pathway (PPP) was observed in response to infection as marked by lower levels of ribulose and sedoheptulose-7-phosphate. Thus, infected hearts have increased glycolysis.

### Tricarboxylic acid cycle (TCA)

**Figure 3:**
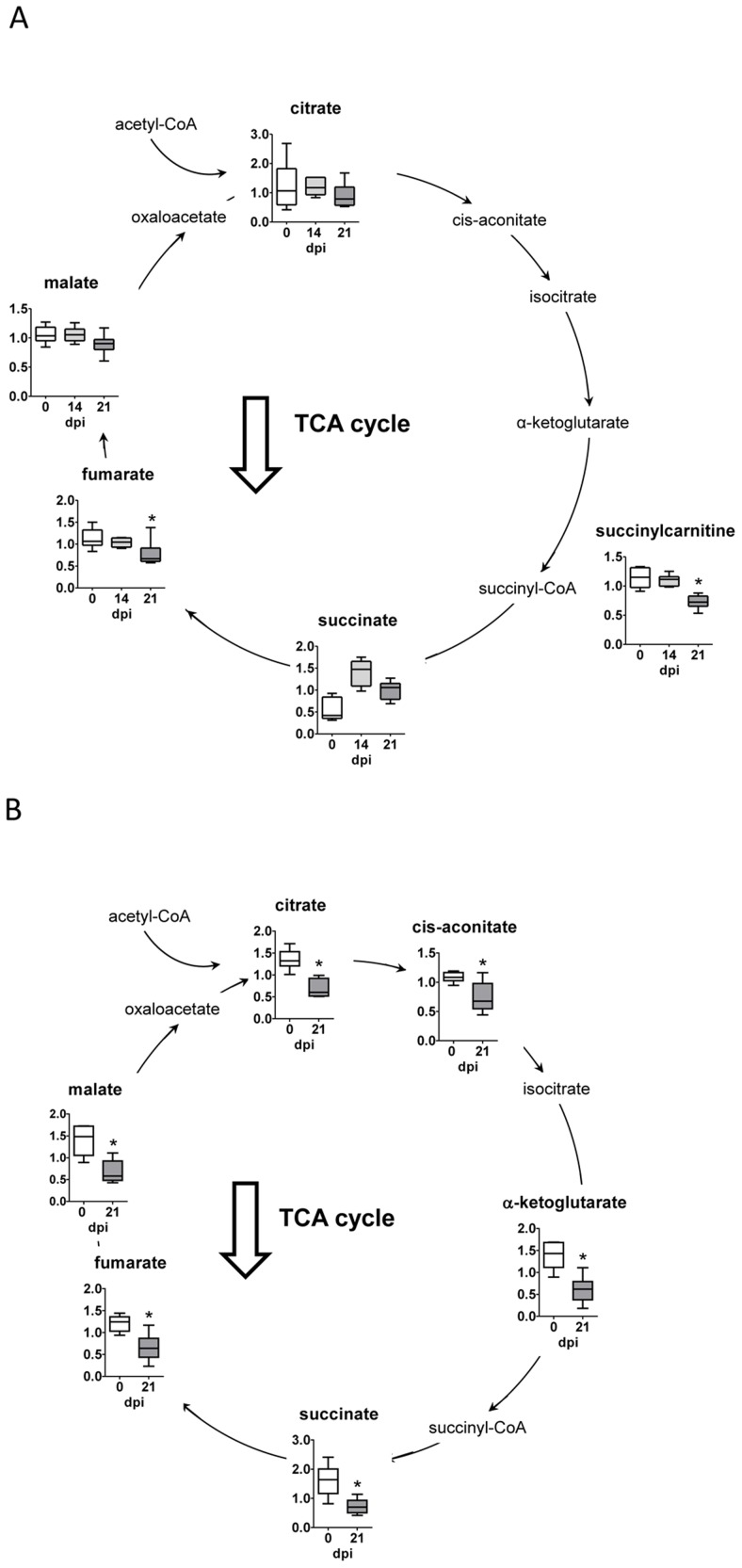
Tricarboxyilic acid cycle. (A) Graphs represent the ScaledImpData of different biochemicals of the tricarboxyilic acid cycle from heart tissue samples. (B) Same as in A from plasma samples. Samples from control uninfected mice are in white boxes, from mice sacrificed at 14 dpi in light gray boxes and from mice sacrificed at 21 dpi in dark grey boxes. Statistically significant differences respect to uninfected mouse samples are denoted, *p≤0.05.

In infected heart tissue, the TCA cycle intermediates, succinylcarnitine and fumarate decreased (Figure 3A). This TCA cycle imbalance in the heart may be indicative of decreased oxidative metabolism, potentially resulting from succinate dehydrogenase (SDH) and electron transport complex II dysfunction. However, all of the TCA cycle metabolites, including succinate, were diminished in plasma of infected mice (Figure 3B).

### Lipid metabolism

**Figure 4:**
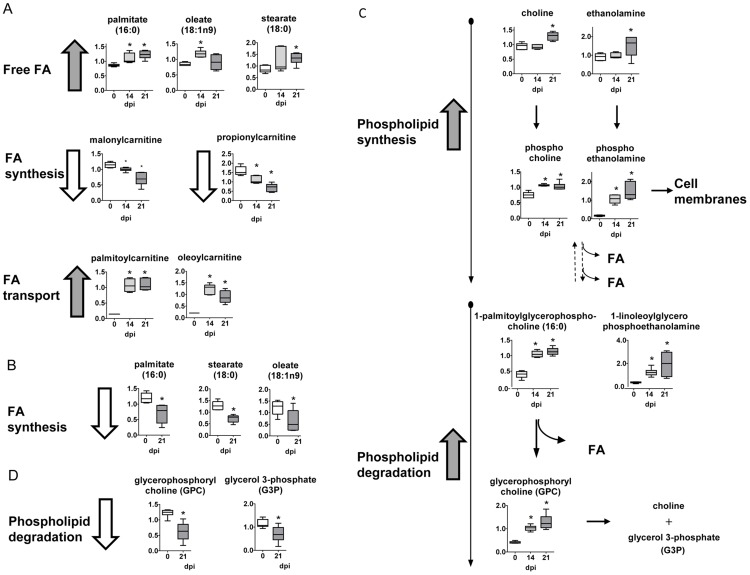
Fatty acid and phospholipid metabolism. (A) Graphs represent the ScaledImpData of different biochemicals of fatty acid metabolism from heart tissue. (B) Same as in A from plasma samples. (C) Same as in A of different biochemicals of phospholipid metabolism from heart tissue samples. (D) Same as in C from plasma samples. Samples from control uninfected mice are in white boxes, from mice sacrificed at 14 dpi in light gray boxes and from mice sacrificed at 21 dpi in dark grey boxes. Statistically significant differences respect to uninfected mouse samples are denoted, *p≤0.05.

Following infection, long chain fatty acids such as palmitate, stearate and oleate increased in heart tissue, but reduced in the plasma (Figure 4A and 4B, respectively). Although changes in fatty acid levels may be indicative of a difference in synthesis, malonylcarnitine levels were significantly diminished in infected tissue, suggesting a limited capacity for lipid biogenesis. Instead, uptake of lipids from plasma and fatty acid β-oxidation in the heart tissue may be altered considering that long chain carnitine conjugate lipids such as palmitoylcarnitine and oleoylcarnitine were much higher in infected animals and may reflect increased lipid transport into the mitochondria, as evidenced by lower levels of carnitine.

### Phospholipid metabolism

The phospholipid catabolite glycerophosphorylcholine (GPC) was elevated in heart tissue after infection (Figure 4C). This may be indicative of a change in phospholipid dynamics since it was accompanied by much higher levels of multiple lysolipids such as 1-linoleoylglycerophosphoethanolamine and 1-palmitoleoylglycerophosphocholine that may reflect enhanced hydrolysis of phospholipids or lipid bodies. In contrast, these metabolites were diminished or unaltered in the plasma of infected animals (Figure 4D). Additionally, phospholipid precursors as choline, choline phosphate, ethanolamine, and phosphoethanolamine especially at late time points also accumulated in infected heart tissue and may be indicative of an increased capacity for phospholipid synthesis, and thus very high accumulation of phospholipids in the heart of infected animals.

### Branched chain amino acid metabolism (BCAA)

**Figure 5:**
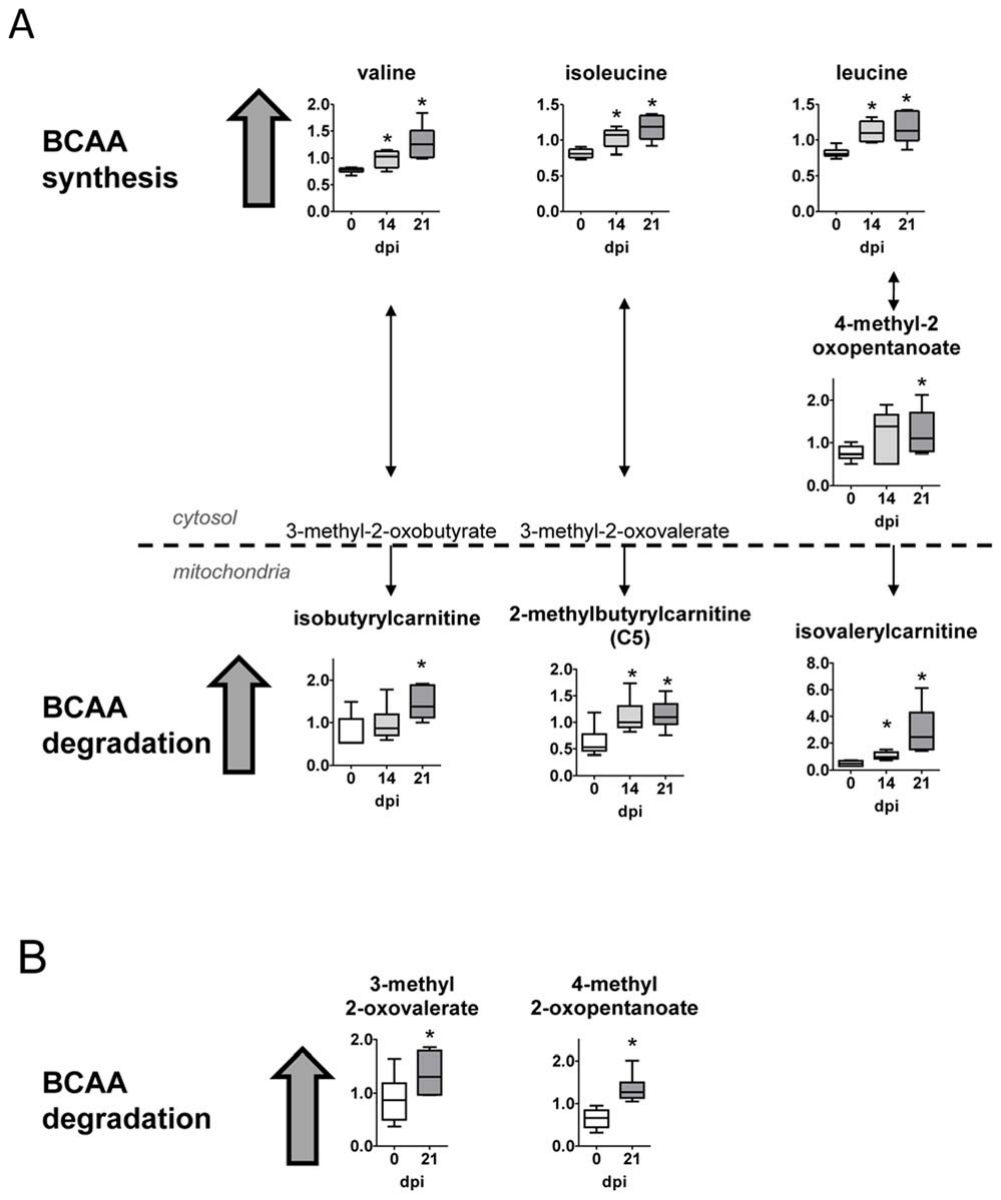
Branched chain amino acid metabolism. (A) Graphs represent the ScaledImpData of different biochemicals of the branched chain amino acid metabolism from heart tissue samples. (B) Same as in A from plasma samples. Samples from control uninfected mice are in white boxes, from mice sacrificed at 14 dpi in light gray boxes and from mice sacrificed at 21 dpi in dark grey boxes. Statistically significant differences respect to uninfected mouse samples are denoted, *p≤0.05.

Heart tissue from *T. cruzi* infected mice possessed higher levels of the BCAAs leucine, isoleucine and valine (Figure 5A). Differences in BCAA levels can reflect changes in their degradation and utilization. In this sense, the downstream products 2-methylbutyrylcarnitine, isovalerylcarnitine and alpha-keto acids (3-methyl-2-oxovalerate and 4-methyl-2-oxopentanoate) were significantly elevated in heart. Additionally, BCAA catabolites including the alpha-keto acids were also increased in plasma (Figure 5B).

### Nucleotide metabolism

**Figure 6:**
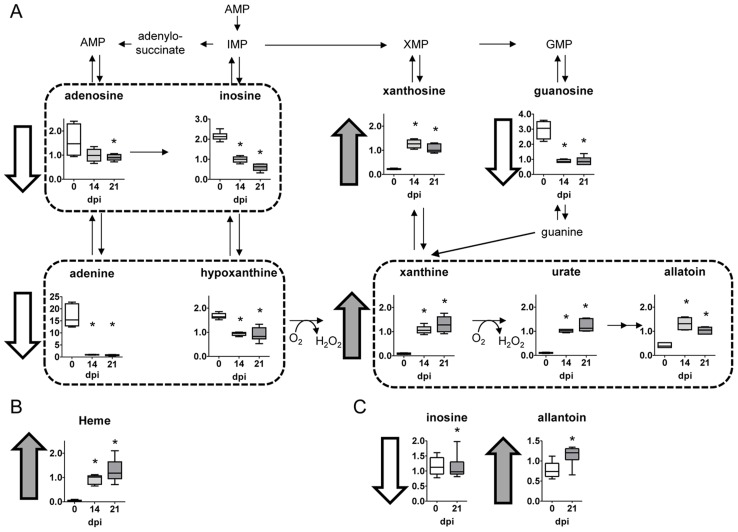
Nucleotide metabolism. (A) Graphs represent the ScaledImpData of different biochemicals of the nucleotide metabolism from heart tissue samples. (B) Same as in A from plasma samples. Samples from control uninfected mice are in white boxes, from mice sacrificed at 14 dpi in light gray boxes and from mice sacrificed at 21 dpi in dark grey boxes. Statistically significant differences respect to uninfected mouse samples are denoted, *p≤0.05.

Compared to uninfected controls, heart tissue from *T. cruzi* infected mice dramatically exhibited much lower levels of multiple purine nucleotides and related metabolites such as adenine, inosine, and hypoxanthine being the levels of adenine almost depleted (Figure 6A). Such metabolites indicate severe alterations in redox homeostasis. In addition, heme, another product of redox activity increased 20–30 times in the infected heart (Figure 6B). In contrast, higher levels of xanthosine, xanthine, urate and allantoin were found in heart tissue from infected mice (Figure 6A). Similar to heart tissue, lower levels of inosine and increased allantoin were found in plasma (Figure 6C). These results may reflect a decrease in synthesis as supported by lower levels of pentose phosphate pathway metabolism. However, more likely purine degradation may be strongly enhanced as evidenced by the great accumulation of the downstream catabolic products xanthosine, urate, and allantoin in infected hearts.

### Tryptophan metabolism

**Figure 7:**
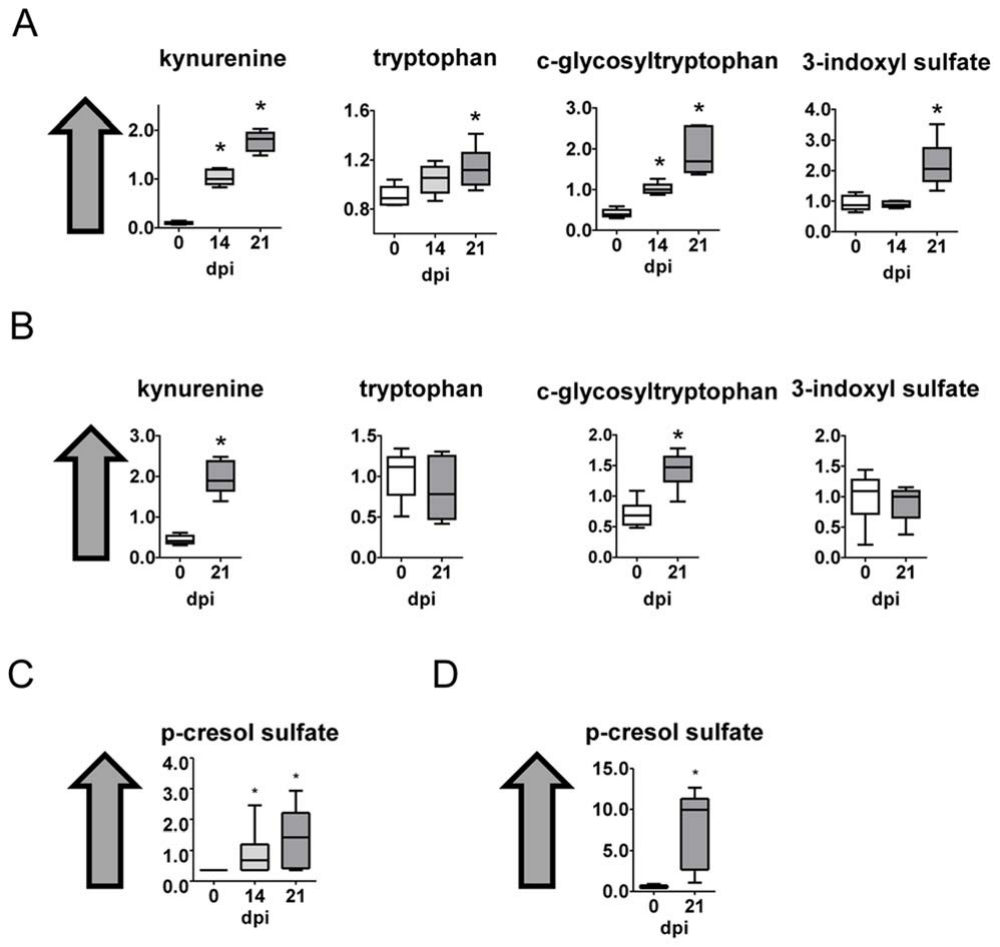
Tryptophan, phenylalanine and tyrosine metabolism. (A) Graphs represent the ScaledImpData of different biochemicals of the tryptophan metabolism from heart tissue samples. (B) Same as in A from plasma samples (C) A Graphs represent the ScaledImpData of different biochemicals of the phenylalanine and tyrosine metabolism from heart tissue samples. (D) Same as in C from plasma samples. Samples from control uninfected mice are in white boxes, from mice sacrificed at 14 dpi in light gray boxes and from mice sacrificed at 21 dpi in dark grey boxes. Statistically significant differences respect to uninfected mouse samples are denoted, *p≤0.05.

Tryptophan and some of its metabolites as kynurenine, c-glycosyltryptophan and 3-indoxyl sulfate were increased in heart tissue (Figure 7A) as well as kynurenine and c-glycosyltryptophan in plasma from infected mice (Figure 7B).

### p-Cresol sulfate

p-Cresol sulfate is part of the phenylalanine and tyrosine metabolism, and it was found strongly increased both in heart tissue and plasma from infected mice (Figure **7C** and **7D**, respectively). p-Cresol sulfate is likely a microbial metabolite that is found in urine and blood and likely derives from secondary metabolism of p-Cresol.

## Discussion

Chagas is a complex disease with acute and chronic phases showing cardiac alterations. Although, the immunopathogenesis is relatively well established, there are still several issues poorly understood. Among those: why a minority of asymptomatic patients become symptomatic after several years?; and which is the reason for the different clinical manifestations?. Besides, few drugs are available to date, which present adverse effects that many patients cannot tolerate. Thus, new drugs are urgently needed, and to achieve that, a deeper knowledge of clinical pathophysiology is required. In this respect, metabolomics may help to better characterize the pathophysiology of the disease as well as to define new biomarkers.

In an unprecedented study, we performed global metabolomic analysis in plasma and heart of mice infected with the *T. cruzi* Y strain. This parasite strain has been considered reticulotropic by many authors in the field. However, other authors claim that the strain might have suffered a change from reticulotropic to myotropic/cardiotropic through time [28]. In agreement with the last, in our experimental model it infects cardiomyocytes [29], produces a characteristic histologic alterations as homogeneous pancarditis with inflammatory infiltrates along epicardium, a high epicardial and sub-epicardial inflammation that was homogenous in auricles and ventricles, myeloid and lymphoid infiltration and intra-myocardial perivasculitis [30] resembling damage observed in Chagasic patients [31]. Moreover, Our analysis revealed that following infection there are many significant biochemical alterations in heart and plasma of infected animals, which are much more evident both in number of changes and in fold variations in the heart, the target organ of *T. cruzi* infection. Our results have identified more than 200 biochemicals (around 2/3) that differed significantly in heart and 100 (around 1/3) in plasma. Some of those showed extremely marked and highly significant differences. More importantly, our results unravel many new aspects of metabolic alterations that can be useful for better understanding the pathogenesis of this disease and to better control *T. cruzi* infection as well as showing the potential of particular metabolites as biomarkers. Our study identifies common biomarkers of cardiomyopathy but more importantly specific candidate biomarkers of acute Chagas Disease Cardiomyopathy, not observed in a clinically similar disease as Idiopathic Dilated Cardiomyopathy.

Alterations include amino acid metabolism, glucose utilization, the TCA cycle, nucleotide catabolism, and membrane lipid pathways. Glucose can be utilized to support a variety of physiological processes including energy generation, fatty acid synthesis, and nucleotide biogenesis. Our results show that compared to control uninfected animals, glucose levels were elevated in heart tissue by days 14 and 21 post-infection. This observation may reflect an increase in glucose uptake considering that glucose levels declined in infected plasma over time. Furthermore, the accumulation of the sorbitol pathway metabolites (sorbitol and fructose) in the heart, but not in plasma, may be an indication of enhanced glucose uptake since excess glucose is often reduced to sorbitol by aldose reductase. Consequently, higher levels of sorbitol can contribute to the generation of advanced glycation end products (AGE) that have been associated with the development of heart failure in other diseases [32]. In addition, multiple glycolytic intermediates including glucose-6 phosphate and fructose-6-phosphate and the end products lactate and pyruvate were significantly elevated in infected heart tissue and may be indicative of increased glycolysis. In this regard, accelerated rates of glycolysis can be indicative of hypertrophy of the heart [33] that accompanies many forms of heart dysfunction, a hallmark of Chagas disease [34]. Thus, these alterations in glycolytic metabolism may be associated with increased cardiac stress in *T. cruzi* infected hearts and require further investigation. In contrast, reduced glucose shuttling to the pentose phosphate pathway (PPP) was observed in response to infection, which is required for the biogenesis of nucleotides and the regeneration of NADPH necessary for glutathione reduction and anabolic reactions. Thus, diminished PPP metabolism may restrict the biosynthetic capacity of the heart and contribute to altered redox homeostasis.

Altered glycolytic metabolism suggested that the TCA cycle may also differ following *T. cruzi* infection. In fact, in *T. cruzi* infected mice an imbalanced TCA cycle in the heart is likely indicative of decreased oxidative metabolism, potentially resulting from succinate dehydrogenase (SDH) and electron transport complex II dysfunction. This would be in agreement with previous studies where chagasic hearts showed mitochondrial respiratory chain impairment [35]. Since the heart has perpetually high energy demands related to the maintenance of processes such as ion transport, calcium homeostasis, and sarcomeric function, the fact that TCA cycle mediated oxidative metabolism may be diminished following infection may ultimately induce metabolic stress in cardiac tissue. In this respect, disruption of the Krebs cycle is often implicated in energetic imbalances characteristic of myocardial ischemia [36], [37]. Alternatively, those selective differences in succinate may be explained by interference of those metabolites by *T. cruzi* metabolism due to fumarate reductase and succinate dehydrogenase enzymatic activities expressed by this parasite [38].

In addition to protein synthesis, branched chain amino acids (BCAA) can be degraded to replenish the TCA cycle and facilitate fatty acid synthesis. Furthermore, diverse injuries and heart diseases are reported to increase consumption of BCAAs. In agreement, heart tissue from *T. cruzi* infected mice possessed higher levels of the BCAAs isoleucine, leucine and valine. Differences in BCAA levels can reflect changes in degradation and amino acid utilization. Additionally, multiple BCAA catabolites including the alpha-keto acids 3-methyl-2-oxovalerate and 4-methyl-2-oxopentanoate and the downstream products 2-methylbutyrylcarnitine and isovalerylcarnitine were significantly elevated in these tissues as well as plasma. Thus, these observations may reflect an increase in amino acid and muscle turnover following infection.

Fatty acids are a critical source of energy for mitochondrial oxidation and cellular ATP generation. Following infection, long chain fatty acids such as palmitate, stearate, and oleate were elevated in heart tissue, but reduced in plasma. Although changes in fatty acid levels may be indicative of a difference in synthesis, malonylcarnitine levels were significantly diminished in infected tissue, suggesting a limited capacity for lipid synthesis. On the contrary, long chain carnitine conjugate lipids such as palmitoylcarnitine and oleoylcarnitine were high suggesting that lipid transport into the mitochondria may be increased. Collectively, these observations suggest that lipid accumulation due to fatty acid uptake in this tissue may not promote intracellular parasite replication by enhancing lipid oxidation as described [24], but instead, by producing a great deal of host cell lipid bodies, a characteristic trait in *T. cruzi* infection [39]. Interestingly, cardiac lipotoxicity results from elevated palmitoly-L-carnitine [40], which we found elevated in hearts of *T. cruzi* infected mice. On the contrary, propionyl-carnitine is considered a cardiac protector [41] and we found it decreased in infected heart.

Phospholipids are essential components of the membrane lipid bilayer. In comparison to control heart tissue, the phospholipid catabolite glycerophosphorylcholine (GPC) was elevated following infection. It may be indicative of a change in phospholipid dynamics and were accompanied by strikingly higher levels of multiple lysolipids such as 1-linoleoylglycerophosphoethanolamine and 1-palmitoleoylglycerophosphocholine that may reflect enhanced hydrolysis of phospholipids or production of lipid bodies. Additionally, the phospholipid precursors choline, choline phosphate, ethanolamine, and phosphoethanolamine also accumulated in infected heart tissue and may be indicative of an increased capacity for phospholipid synthesis. Differences in these metabolites may reflect tissue remodeling during infection.

Noteworthy, heart tissue from *T. cruzi* infected mice was almost depleted of adenine and had much lower levels of purine nucleotides and metabolites such as adenosine, guanosine, inosine, and hypoxanthine, compared to uninfected controls. Lower levels of these metabolites may reflect a decrease in synthesis as supported by lower levels of pentose phosphate pathway metabolism. But more likely, purine degradation may be enhanced as evidenced by a strong accumulation of the downstream catabolic products xanthosine, xanthine, urate, and allantoin in infected hearts. Differences in the levels of these metabolites may also reflect a change in redox homeostasis since the generation of xanthine and urate are accompanied by the production of hydrogen peroxide (H_2_O_2_). Thus, differences in purine metabolism may contribute to altered redox homeostasis in cardiac tissue. In addition, it has been described that the parasite scavenges purines and pteridine for amastigote replication in HeLa cells [24]. Thus, the decreased levels of adenine observed in heart tissue may reflect both phenomena: decreased synthesis and parasite utilization.

Interestingly, xantine oxidase (XOS) the enzyme responsible for reactive oxygen species (ROS) generation has been considered responsible for cardiac dysfunction in *T. cruzi* infection [42]. Thus, allopurinol, a xanthine oxidase inhibitor, is being considered for treating Chagasic patients [43]. Inhibition of ROS generation ameliorates myocarditis during Chagas disease, although parasite burden in the heart was not significantly decreased [44]. Interestingly, the effect of ROS inhibition seems to be related with the levels of metabolites as substrates of immune-related enzymes and thus their imbalances can be targeted to combat infectious diseases.

Besides, the fact that uric acid is so strongly elevated and adenine so depleted may indicate that ROS production in the heart should be very high (Figure 6A). ROS also increase the ability of *T. cruzi* to replicate intracellularly [45]. Uric acid is linked to several cardiovascular diseases [46], [47], [48], although there is still discrepancy on whether it is really the cause or the effect of the disease [49]. The increase in uric acid is also associated to elevated fructose [50], also shown in our analysis. Uric acid is able to activate inflammasome NLRP3, being a danger signal in many pathological processes and in inflammation. Higher signaling through NLRP3 has been recently related to adverse outcome in other forms of cardiac dysfunction as idiopathic dilated cardiomyopathy [51]. However, in apparent contrast, it has been recently demonstrated a protective role of NLRP3 inflammasome in the control of *T.cruzi* infection through caspase-1-dependente IL-1R-independent nitric oxide (NO) production [52]. Thus, it would be worth testing the role of NLRP3 in Chagas disease cardiomyopathy and not only relative to infection.

In addition, Heme, a product involved in redox homeostasis, was increased in infected heart, and is also able to increase *T. cruzi* growth [53]. Increased heme levels are found in failing hearts [54] associated to ROS production. In this regard, it is also worth mentioning that allopurinol, that inhibits XOS, has been used effectively against *T. cruzi* in mouse models [43], [55] and also to prevent cardiovascular dysfunction (CVD) [48].

Lysoglycerol-phosphocholines, that increased very significantly in *T. cruzi* infected heart, may also contribute to immunosuppression associated to Chagas disease [56], since they inhibit T-cell proliferation in response to activation and induce apoptosis [57].

Kynurenine is not usually found in heart tissue, and acts as an endothelium-derived relaxing factor produced during inflammation [58]. It can also control dendritic cell immunogenicity [59]. Notably, this metabolite has been implicated in resistance to *T. cruzi* infection [19], [20]. Furthermore, the serum levels of IDO enzymatic activity in Chagas disease symptomatic patients decrease with therapeutic treatment [60]. Since, IDO activity is often elevated in response to inflammatory cytokines such as TNFα and IFNγ, elevated in cardiac patients [12], these findings may reflect immune cell activation in response to *T. cruzi* infection.

p-Cresol sulfate and indoxyl sulfate are considered uremic toxins [61]. Although they are generally associated to dialysis disturbances they have an emerging role in cardiovascular disease and mortality in renal patients. Serum p-Cresol sulfate predicts cardiovascular disease and mortality in elderly hemodialysis patients [62]. In patients with chronic renal failure p-cresol sulphate accumulation in plasma is due to renal dysfunction and cause endothelial dysfunction increasing the cardiovascular risk [63]. We found it highly increased in heart tissue as well as serum of infected mice. The levels of p-cresol sulphate can be also predictive of clinical outcome. Our work, if confirmed in Chagas disease patients, may suggest that p-cresol sulphate could be useful a marker of *T. cruzi* infection and further studies are needed to check if it also correlates with disease outcome.

Recently, a global metabolomic analysis in an experimental hamster model of idiopathic dilated cardiomyopathy (DCM) has been described [14]. By comparison with our data some specific biomarkers, and by extension pathogenic mechanism(s), can be inferred, that can help to distinguish among those clinically similar but etiologically distinct dilated cardiomyopathies. In that study, only 180 metabolites were detected and variations were found in 62 of them. Their results suggest that the glycolysis and the TCA cycle energy pathways are attenuated in cardiac tissue during the symptomatic phase. In contrast, hearts from *T. cruzi* infected mice have increased glycolysis, despite having the TCA cycle decreased. Similar to our results they observed increased oxidative stress in the DCM. Thus, the rest of alterations we found can be considered specific, so far, of cardiomyopathy associated to *T. cruzi* infection. Among those, much higher lipid (and specially phospholipids) accumulation in *T. cruzi* infected hearts as well as stronger depletion of adenine and increase in allantoin and uric acid.

Previous studies on metabolic alterations caused by *T. cruzi* infection were based only on host enzymatic expression *in vitro* [24]. Thus our work greatly expands those results by detecting their metabolites *in vivo* in a mouse model of infection. Our findings suggest that *T. cruzi* infection disrupts multiple biochemical pathways in heart tissue that may ultimately contribute to cardiac failure. In particular, increased glycolysis and decreased oxidative metabolism may predispose heart muscle to energetic imbalances characteristic of heart failure [64]. Furthermore, altered glucose utilization may limit the anabolic capacity of the heart and therefore the ability to repair damaged tissue and to detoxify free radicals. Consequently, evidence of altered redox homeostasis, inflammation, and tissue remodeling were observed in response to infection.

In summary, we have found many metabolic alterations in experimental acute Chagas disease. Many of these suggest a stressful condition in the heart such as: a) increased glycolysis, b) respiratory chain impairment, c) lipid accumulation, d) ROS production and uric acid formation. All of those could be related with heart hypertrophy. Noteworthy, some of those metabolites have much stronger variations than reported in other cardiac pathologies. Probably more interestingly, we found elevated p-cresol sulfate, which is associated to infections, and kynurenine and allantoin in serum that, either individually or in combination, may be specific biomarkers in this disease. Moreover, p-cresol sulfate, kynurenine and allantoin presented very high positive coefficients in the PCA analysis in plasma reinforcing its value as possible biomarkers. Very importantly, in the evaluation of new drugs against Chagas disease, a biomarker of cardiac function recovery is urgently needed to assess the efficacy of new treatments in the clinic-pathological symptoms and not only for determining parasitological cure. Despite that our study spans only the acute Chagas disease, taking into account that acute and chronic phases share some similarities, the possibility that any of the candidate biomarkers may apply to the chronic phase cannot be excluded. Thus, future studies may benefit from examining metabolic differences induced by acute versus chronic infection to determine the long-term risk factors associated with *T. cruzi* infection, as well as exploring the usefulness of the ones described here as biomarkers of clinical improvement.

## Supporting Information

Table S1 **Summary of the significantly altered biochemicals.** 325 biochemical were identified in heart extracts and 306 in plasma extract. Following log transformation and imputation with minimum observed values for each compound, Welch's two-sample t-test was used to identify biochemicals that differed significantly between experimental groups. Biochemicals that achieved statistical significance (p≤0.05), as well as those approaching significance (0.05<p<0.10), is shown. Increased levels of biochemicals are in red and decreased levels in green.
(DOCX)

Table S2 **Contribution of the heart tissue individual biochemicals to the Principal components analysis (PCA).** List of the calculated coefficients of heart tissue biochemicals ordered from higher to lower. The higher positive and negative coefficients are the ones that have more contribution for the PCA analysis. Component 1 may have the greatest contribution to separating the metabolic signature followed by component 2. Plasma candidate biomarkers as p-cresol sulphate, kynurenine and allantoin, which increase with the infection are highlighted.
(DOCX)

Table S3 **Contribution of the plasma individual biochemicals to the Principal components analysis (PCA).** List of the calculated coefficients of heart tissue biochemicals ordered from higher to lower. The higher positive and negative coefficients are the ones that have more contribution for the PCA analysis. Component 1 may have the greatest contribution to separating the metabolic signature followed by component 2. Plasma candidate biomarkers as p-cresol sulphate, kynurenine and allantoin, which increase with the infection, with very high positive coefficients are highlighted.
(DOCX)
