# Supplementary material for: Global Metabolomic Profiling of Acute Myocarditis Caused by Trypanosoma cruzi Infection
Source: PLoS Negl Trop Dis. 2014 Nov 20;8(11):e3337. doi: 10.1371/journal.pntd.0003337 (PMC4239010; doi:10.1371/journal.pntd.0003337)
Supplement: Table S1 — Summary of the significantly altered biochemicals. 325 biochemical were identified in heart extracts and 306 in plasma extract. Following log transformation and imputation with minimum observed values for each compound, Welch's two-sample t-test was used to identify biochemicals that differed significantly between experimental groups. Biochemicals that achieved statistical significance (p≤0.05), as well as those approaching significance (0.05<p<0.10), is shown. Increased levels of biochemicals are in red and decreased levels in green. (DOCX) [file pntd.0003337.s001.docx]

**Table S1.**

| **Statistical Comparisons Welch's Two-Sample t-Test** | | | | |
| --- | --- | --- | --- | --- |
| ***Significantly Altered Biochemicals*** | **Heart Extract** | | | **Plasma Extract** |
|  | **Infected (14 dpi) Non-Infected** | **Infected (21 dpi) Non-Infected** | **Infected (21 dpi) Infected (14 dpi)** | **Infected (21 dpi) Non-Infected** |
| Total biochemicals *p*≤0.05 | 202 | 217 | 101 | 100 |
| Biochemicals  (↑↓) | 170\|32 | 174\|43 | 51\|50 | 22\|78 |
| Total biochemicals 0.05<*p*<0.10 | 19 | 18 | 33 | 41 |
| Biochemicals  (↑↓) | 16\|3 | 10\|8 | 21\|12 | 6\|35 |
