# Supplementary material for: Global Metabolomic Profiling of Acute Myocarditis Caused by Trypanosoma cruzi Infection
Source: PLoS Negl Trop Dis. 2014 Nov 20;8(11):e3337. doi: 10.1371/journal.pntd.0003337 (PMC4239010; doi:10.1371/journal.pntd.0003337)
Supplement: Table S2 — Contribution of the heart tissue individual biochemicals to the Principal components analysis (PCA). List of the calculated coefficients of heart tissue biochemicals ordered from higher to lower. The higher positive and negative coefficients are the ones that have more contribution for the PCA analysis. Component 1 may have the greatest contribution to separating the metabolic signature followed by component 2. Plasma candidate biomarkers as p-cresol sulphate, kynurenine and allantoin, which increase with the infection are highlighted. (DOCX) [file pntd.0003337.s002.docx]

**Table S2.**

|  | **Biochemical** | **Comp.1 [44.21%]** | **Comp.2 [12.08%]** |
| --- | --- | --- | --- |
| 1 | N-acetylneuraminate | 0,0817 | 0,0109 |
| 2 | assymetric dimethylarginine (ADMA) | 0,0815 | 0,0072 |
| 3 | homocysteine | 0,0813 | 0,0256 |
| 4 | thymine | 0,0813 | 0,0245 |
| 5 | itaconate (methylenesuccinate) | 0,081 | 0,0306 |
| 6 | guanosine 5'- monophosphate (5'-GMP) | 0,0808 | 0,0084 |
| 7 | gulono-1,4-lactone | 0,0806 | -0,006 |
| 8 | phosphoethanolamine | 0,0804 | -0,0005 |
| 9 | urate | 0,0802 | -0,0278 |
| 10 | glycerophosphorylcholine (GPC) | 0,0798 | -0,0228 |
| 11 | C-glycosyltryptophan* | 0,0793 | 0,04 |
| 12 | 1-myristoylglycerophosphocholine (14:0) | 0,0792 | 0,0056 |
| 13 | N-acetylserine | 0,0789 | -0,0107 |
| 14 | N-glycolylneuraminate | 0,0789 | 0,0364 |
| 15 | 1-arachidonoylglycerophosphoethanolamine* | 0,0787 | 0,0047 |
| 16 | 2-oleoylglycerophosphoserine* | 0,0786 | 0,0258 |
| 17 | 2-palmitoylglycerophosphocholine* | 0,0784 | -0,0416 |
| 18 | 1-palmitoylglycerophosphoinositol* | 0,0782 | -0,0016 |
| 19 | 2'-deoxycytidine | 0,0782 | 0,0364 |
| 20 | pyridoxate | 0,078 | -0,0215 |
| 21 | 1-arachidonoylglycerophosphoinositol* | 0,0779 | 0,0024 |
| 22 | 1-linoleoylglycerophosphoethanolamine* | 0,0778 | 0,0358 |
| 23 | 1-oleoylglycerophosphocholine (18:1) | 0,0777 | -0,0452 |
| 24 | 2-oleoylglycerophosphoethanolamine* | 0,0777 | 0,003 |
| 25 | proline | 0,0777 | 0,0392 |
| 26 | glycine | 0,0775 | -0,002 |
| 27 | gluconate | 0,0772 | 0,0155 |
| 28 | pipecolate | 0,0771 | 0,0285 |
| 29 | 1-linoleoylglycerophosphocholine (18:2n6) | 0,077 | -0,0371 |
| 30 | 2-arachidonoylglycerophosphocholine* | 0,0769 | -0,0435 |
| 31 | 1-palmitoylplasmenylethanolamine* | 0,0765 | -0,0339 |
| 32 | 1-oleoylglycerophosphoserine | 0,0764 | 0,0361 |
| 33 | 2-oleoylglycerophosphocholine* | 0,0764 | -0,0498 |
| 34 | glycerol 2-phosphate | 0,0763 | -0,0072 |
| 35 | 2-linoleoylglycerophosphoethanolamine* | 0,0761 | 0,0377 |
| 36 | kynurenine | 0,0761 | -0,0072 |
| 37 | 1-linoleoylglycerophosphoinositol* | 0,0757 | -0,0049 |
| 38 | N4-acetylcytidine | 0,0757 | 0,0414 |
| 39 | N6-acetyllysine | 0,0755 | 0,0522 |
| 40 | 2-arachidonoylglycerophosphoethanolamine* | 0,0754 | 0,016 |
| 41 | 2-linoleoylglycerophosphocholine* | 0,0754 | 0,0121 |
| 42 | fucose | 0,0754 | 0,0259 |
| 43 | inositol 1-phosphate (I1P) | 0,0751 | 0,0355 |
| 44 | 1-docosahexaenoylglycerol (1-monodocosahexaenoin) | 0,075 | 0,02 |
| 45 | galactose | 0,075 | 0,0236 |
| 46 | palmitate (16:0) | 0,0748 | -0,0012 |
| 47 | 1-arachidonylglycerol | 0,0746 | 0,0237 |
| 48 | ornithine | 0,0746 | 0,0014 |
| 49 | 1-dihomo-linoleoylglycerophosphocholine (20:2n6)* | 0,074 | 0,0248 |
| 50 | 1-oleoylglycerophosphoethanolamine | 0,074 | -0,0101 |
| 51 | glutamate, gamma-methyl ester | 0,0739 | -0,0359 |
| 52 | 1-linoleoylglycerophosphoserine* | 0,0732 | 0,0628 |
| 53 | 1-oleoylglycerophosphoinositol* | 0,0732 | 0,0193 |
| 54 | 2'-deoxyadenosine 3'-monophosphate | 0,0731 | -0,0676 |
| 55 | methionine | 0,0731 | 0,0114 |
| 56 | erythronate* | 0,073 | -0,0002 |
| 57 | 2-docosahexaenoylglycerophosphocholine* | 0,0728 | -0,066 |
| 58 | 2-linoleoylglycerophosphoinositol* | 0,0728 | -0,0394 |
| 59 | tyrosine | 0,0725 | 0,009 |
| 60 | isoleucine | 0,0719 | 0,0094 |
| 61 | adenosine 3'-monophosphate (3'-AMP) | 0,0718 | 0,0517 |
| 62 | 2-palmitoleoylglycerophosphocholine* | 0,0715 | 0,0006 |
| 63 | serine | 0,0713 | 0,0127 |
| 64 | cytidine | 0,0712 | 0,0271 |
| 65 | alpha-hydroxyisovalerate | 0,0711 | 0,0236 |
| 66 | 1-margaroylglycerophosphocholine (17:0) | 0,071 | -0,0292 |
| 67 | uracil | 0,0709 | 0,0052 |
| 68 | palmitoylcarnitine | 0,0708 | -0,0624 |
| 69 | leucine | 0,0706 | -0,003 |
| 70 | N-acetylalanine | 0,0706 | 0,0242 |
| 71 | methionine sulfoxide | 0,0702 | 0,0147 |
| 72 | guanosine 3'-monophosphate (3'-GMP) | 0,0699 | 0,0614 |
| 73 | threonine | 0,0696 | 0,045 |
| 74 | valine | 0,0694 | 0,0488 |
| 75 | sphingosine | 0,0693 | -0,0077 |
| 76 | gamma-glutamylglutamate | 0,0691 | 0,0528 |
| 77 | heme | 0,0689 | -0,0219 |
| 78 | phenol sulfate | 0,0689 | 0,0131 |
| 79 | 1-palmitoylglycerophosphocholine (16:0) | 0,0685 | -0,0642 |
| 80 | 1-stearoylglycerophosphoglycerol | 0,0684 | -0,061 |
| 81 | glycerate | 0,0684 | -0,0424 |
| 82 | erythritol | 0,0683 | -0,0701 |
| 83 | mannose | 0,0683 | 0,0053 |
| 84 | 1-eicosatrienoylglycerophosphocholine (20:3)* | 0,0681 | -0,0704 |
| 85 | xanthine | 0,0678 | -0,0466 |
| 86 | 1-eicosapentaenoylglycerophosphocholine (20:5n3)* | 0,0676 | 0,0632 |
| 87 | stearate, methyl ester | 0,0676 | 0,0472 |
| 88 | putrescine | 0,0675 | -0,069 |
| 89 | 2-arachidonoylglycerophosphoinositol* | 0,0674 | -0,0561 |
| 90 | docosadienoate (22:2n6) | 0,0674 | 0,022 |
| 91 | 2-arachidonoyl glycerol | 0,0673 | -0,0076 |
| 92 | xanthosine | 0,0673 | -0,0863 |
| 93 | 5-methyluridine (ribothymidine) | 0,0672 | 0,0038 |
| 94 | tryptophan | 0,0671 | 0,0101 |
| 95 | arachidonate (20:4n6) | 0,067 | -0,0073 |
| 96 | ergothioneine | 0,0668 | 0,0533 |
| 97 | gamma-glutamylleucine | 0,0668 | 0,0505 |
| 98 | isovalerylcarnitine | 0,0664 | 0,0805 |
| 99 | cytidine 5'-monophosphate (5'-CMP) | 0,0663 | -0,0543 |
| 100 | dihomo-linoleate (20:2n6) | 0,0661 | 0,0233 |
| 101 | beta-alanine | 0,0659 | -0,0051 |
| 102 | phenylacetylglycine | 0,0656 | 0,039 |
| 103 | dimethylglycine | 0,0654 | -0,0845 |
| 104 | asparagine | 0,065 | -0,0012 |
| 105 | 2-linoleoylglycerol (2-monolinolein) | 0,0648 | -0,0286 |
| 106 | fructose | 0,0648 | 0,0031 |
| 107 | 1-palmitoleoylglycerophosphocholine (16:1)* | 0,0644 | -0,0112 |
| 108 | phenylalanine | 0,0643 | -0,0261 |
| 109 | 2-docosahexaenoylglycerophosphoethanolamine* | 0,0642 | -0,0816 |
| 110 | pro-hydroxy-pro | 0,064 | 0,066 |
| 111 | Isobar: fructose 1,6-diphosphate, glucose 1,6-diphosphate, myo-inositol 1,4 or 1,3-diphosphate | 0,0638 | -0,017 |
| 112 | cysteine-glutathione disulfide | 0,0636 | 0,0657 |
| 113 | 6-keto prostaglandin F1alpha | 0,0632 | -0,021 |
| 114 | dehydroascorbate | 0,0623 | -0,0122 |
| 115 | lysine | 0,0623 | 0,0477 |
| 116 | trans-urocanate | 0,0621 | 0,0976 |
| 117 | glutamate | 0,0616 | 0,0075 |
| 118 | glutathione, oxidized (GSSG) | 0,0612 | 0,0351 |
| 119 | N-delta-acetylornithine* | 0,061 | 0,0575 |
| 120 | N-acetylthreonine | 0,0609 | -0,0227 |
| 121 | allantoin | 0,0608 | -0,0793 |
| 122 | adenosine 2'-monophosphate (2'-AMP) | 0,0603 | -0,0671 |
| 123 | glucosamine | 0,0603 | 0,065 |
| 124 | N-acetylmethionine | 0,0601 | -0,0914 |
| 125 | galactitol (dulcitol) | 0,0599 | -0,1069 |
| 126 | urea | 0,0594 | 0,0421 |
| 127 | glucose 1-phosphate | 0,0592 | -0,0443 |
| 128 | linoleate, methyl ester | 0,059 | 0,0467 |
| 129 | gamma-glutamylphenylalanine | 0,0589 | 0,0546 |
| 130 | prostaglandin E2 | 0,0589 | 0,0661 |
| 131 | 7-methylguanine | 0,0588 | -0,0203 |
| 132 | fructose-6-phosphate | 0,0587 | -0,0536 |
| 133 | cysteine | 0,0586 | 0,0334 |
| 134 | 2-palmitoylglycerophosphoethanolamine* | 0,0583 | -0,0041 |
| 135 | p-cresol sulfate | 0,0583 | 0,0427 |
| 136 | 2'-deoxyuridine | 0,058 | 0,0191 |
| 137 | riboflavin (Vitamin B2) | 0,0577 | -0,0119 |
| 138 | 2'-deoxycytidine 5'-monophosphate | 0,0576 | -0,104 |
| 139 | 1-docosapentaenoylglycerophosphocholine (22:5)* | 0,0574 | -0,0994 |
| 140 | alanine | 0,0573 | 0,0033 |
| 141 | N2,N2-dimethylguanosine | 0,0572 | 0,0761 |
| 142 | 1-linoleoylglycerol (1-monolinolein) | 0,057 | -0,0097 |
| 143 | N-acetylaspartate (NAA) | 0,0564 | 0,0529 |
| 144 | glucose | 0,0562 | 0,0272 |
| 145 | 3-(4-hydroxyphenyl)lactate | 0,0559 | 0,0823 |
| 146 | choline phosphate | 0,0554 | -0,0928 |
| 147 | choline | 0,0552 | 0,0829 |
| 148 | 2-methylbutyrylcarnitine (C5) | 0,0551 | -0,0375 |
| 149 | N6,N6-dimethyladenosine | 0,0548 | 0,0301 |
| 150 | 5-methylcytidine | 0,0547 | 0,0434 |
| 151 | sphinganine | 0,0543 | -0,0211 |
| 152 | 2'-deoxyinosine | 0,0536 | -0,116 |
| 153 | coenzyme A | 0,0536 | 0,0873 |
| 154 | 3-indoxyl sulfate | 0,0534 | 0,0923 |
| 155 | stearate (18:0) | 0,053 | -0,0138 |
| 156 | 4-methyl-2-oxopentanoate | 0,0524 | 0,0194 |
| 157 | xylonate | 0,051 | -0,0243 |
| 158 | oleoylcarnitine | 0,0505 | -0,1042 |
| 159 | ophthalmate | 0,0501 | -0,0677 |
| 160 | purine | 0,0501 | -0,0697 |
| 161 | mannose-6-phosphate | 0,0492 | -0,0584 |
| 162 | sorbitol | 0,0492 | -0,0218 |
| 163 | isobutyrylcarnitine | 0,0491 | 0,017 |
| 164 | margarate (17:0) | 0,0486 | 0,0617 |
| 165 | xylose | 0,0485 | -0,0151 |
| 166 | 1-eicosenoylglycerophosphocholine (20:1n9)* | 0,0484 | 0,0338 |
| 167 | glucose-6-phosphate (G6P) | 0,0484 | -0,0692 |
| 168 | 2-stearoylglycerophosphocholine* | 0,0473 | -0,1173 |
| 169 | pinitol | 0,0451 | 0,0082 |
| 170 | erlose | 0,0449 | 0,0708 |
| 171 | 2-aminobutyrate | 0,0447 | -0,0232 |
| 172 | trans-4-hydroxyproline | 0,0443 | -0,0141 |
| 173 | sarcosine (N-Methylglycine) | 0,0441 | -0,0309 |
| 174 | hydroxyisovaleroyl carnitine | 0,0432 | -0,0224 |
| 175 | 1-arachidonoylglycerophosphocholine (20:4n6)* | 0,0429 | -0,0859 |
| 176 | 1-stearoylglycerophosphocholine (18:0) | 0,0417 | -0,0207 |
| 177 | corticosterone | 0,0415 | 0,0761 |
| 178 | succinate | 0,0415 | -0,104 |
| 179 | N-acetylglycine | 0,0414 | -0,0217 |
| 180 | glycolate (hydroxyacetate) | 0,041 | -0,0956 |
| 181 | linoleate (18:2n6) | 0,0402 | -0,0443 |
| 182 | sucrose | 0,04 | 0,0527 |
| 183 | ethanolamine | 0,0399 | -0,0033 |
| 184 | 5,6-dihydrouracil | 0,039 | -0,1093 |
| 185 | aspartate | 0,0386 | 0,0037 |
| 186 | glycerol 3-phosphate (G3P) | 0,0371 | 0,0227 |
| 187 | oleate (18:1n9) | 0,037 | -0,0476 |
| 188 | stearoylcarnitine | 0,0353 | -0,1071 |
| 189 | campesterol | 0,0344 | 0,0445 |
| 190 | myo-inositol | 0,0343 | -0,07 |
| 191 | deoxycarnitine | 0,0339 | 0,0756 |
| 192 | spermidine | 0,033 | 0,0461 |
| 193 | beta-hydroxypyruvate | 0,0313 | 0,0344 |
| 194 | gamma-aminobutyrate (GABA) | 0,0305 | 0,0149 |
| 195 | 1,3-dihydroxyacetone | 0,0299 | 0,0401 |
| 196 | arabitol | 0,0297 | 0,1075 |
| 197 | 17-methylstearate | 0,0279 | -0,0079 |
| 198 | pseudouridine | 0,0269 | 0,1135 |
| 199 | ribitol | 0,0265 | -0,1132 |
| 200 | mannitol | 0,0262 | -0,1428 |
| 201 | thymidine | 0,0256 | -0,1271 |
| 202 | N6-carbamoylthreonyladenosine | 0,0252 | 0,0173 |
| 203 | hypotaurine | 0,023 | 0,0451 |
| 204 | 2-hydroxybutyrate (AHB) | 0,0228 | -0,0698 |
| 205 | catechol sulfate | 0,0228 | 0,0102 |
| 206 | adenosine 5'-monophosphate (AMP) | 0,0219 | -0,038 |
| 207 | pyruvate | 0,0212 | -0,0838 |
| 208 | N1-methyladenosine | 0,0199 | 0,0503 |
| 209 | ribose 5-phosphate | 0,0196 | -0,0382 |
| 210 | 2'-deoxyguanosine | 0,0183 | -0,0344 |
| 211 | 3-methyl-2-oxovalerate | 0,0179 | 0,0275 |
| 212 | hippurate | 0,0178 | -0,0646 |
| 213 | 1-docosahexaenoylglycerophosphocholine (22:6n3)* | 0,0177 | -0,0962 |
| 214 | 10-heptadecenoate (17:1n7) | 0,0171 | 0,0555 |
| 215 | N1-methylguanosine | 0,0171 | -0,0045 |
| 216 | palmitoleate (16:1n7) | 0,0164 | -0,1109 |
| 217 | taurocholate | 0,0164 | -0,0606 |
| 218 | pyroglutamine* | 0,0155 | -0,0126 |
| 219 | palmitoyl sphingomyelin | 0,0152 | -0,0053 |
| 220 | 3-ureidopropionate | 0,0151 | -0,123 |
| 221 | S-lactoylglutathione | 0,015 | -0,0168 |
| 222 | tauro(alpha + beta)muricholate | 0,0139 | -0,0653 |
| 223 | N-formylmethionine | 0,0123 | -0,0898 |
| 224 | glycylleucine | 0,0119 | -0,0322 |
| 225 | 1-nonadecanoylglycerophosphocholine(19:0)* | 0,0114 | -0,0805 |
| 226 | hydroxybutyrylcarnitine* | 0,0114 | -0,1298 |
| 227 | cis-vaccenate (18:1n7) | 0,011 | 0,0181 |
| 228 | 5-oxoproline | 0,0109 | 0,0753 |
| 229 | chiro-inositol | 0,0105 | 0,0117 |
| 230 | N-acetyl-aspartyl-glutamate (NAAG) | 0,0103 | -0,0743 |
| 231 | glutamine | 0,0101 | 0,0558 |
| 232 | stearoyl ethanolamide | 0,0092 | -0,0709 |
| 233 | homoserine | 0,0079 | -0,0549 |
| 234 | 1,5-anhydroglucitol (1,5-AG) | 0,0077 | 0,0755 |
| 235 | anserine | 0,0072 | -0,0266 |
| 236 | Isobar: ribulose 5-phosphate, xylulose 5-phosphate | 0,0069 | -0,0715 |
| 237 | N-palmitoyl taurine | 0,0069 | -0,0915 |
| 238 | butyrylcarnitine | 0,006 | -0,0462 |
| 239 | docosahexaenoate (DHA; 22:6n3) | 0,0057 | 0,0117 |
| 240 | oleoyltaurine | 0,0056 | -0,1138 |
| 241 | maltose | 0,005 | -0,0505 |
| 242 | lactate | 0,003 | -0,0972 |
| 243 | 4-guanidinobutanoate | 0,0011 | -0,0139 |
| 244 | 10-nonadecenoate (19:1n9) | -0,0001 | -0,0265 |
| 245 | 3-hydroxydecanoate | -0,0013 | -0,1219 |
| 246 | 1-palmitoylglycerophosphoethanolamine | -0,002 | -0,0391 |
| 247 | acetylcarnitine | -0,0026 | -0,1096 |
| 248 | prolylglycine | -0,0028 | -0,0488 |
| 249 | stachydrine | -0,0036 | 0,0287 |
| 250 | creatinine | -0,0039 | -0,1338 |
| 251 | pyrophosphate (PPi) | -0,0052 | -0,0818 |
| 252 | 3-hydroxybutyrate (BHBA) | -0,0058 | -0,0998 |
| 253 | phosphate | -0,006 | -0,0693 |
| 254 | ribose | -0,0103 | -0,021 |
| 255 | 7-alpha-hydroxycholesterol | -0,0109 | 0,0284 |
| 256 | docosapentaenoate (n3 DPA; 22:5n3) | -0,0115 | -0,083 |
| 257 | caprylate (8:0) | -0,0143 | -0,0439 |
| 258 | 7-beta-hydroxycholesterol | -0,0144 | 0,0017 |
| 259 | flavin adenine dinucleotide (FAD) | -0,016 | -0,1374 |
| 260 | 4-hydroxybutyrate (GHB) | -0,0173 | 0,0184 |
| 261 | taurine | -0,0187 | -0,1153 |
| 262 | caprate (10:0) | -0,0202 | -0,092 |
| 263 | azelate (nonanedioate) | -0,0211 | -0,0379 |
| 264 | stearidonate (18:4n3) | -0,0222 | -0,0766 |
| 265 | arginine | -0,0228 | 0,0487 |
| 266 | myristoleate (14:1n5) | -0,0231 | -0,1071 |
| 267 | pantothenate | -0,0232 | -0,0149 |
| 268 | xylitol | -0,0235 | -0,0829 |
| 269 | malate | -0,0243 | -0,0713 |
| 270 | nicotinamide adenine dinucleotide (NAD+) | -0,0257 | -0,0611 |
| 271 | laurate (12:0) | -0,026 | -0,0937 |
| 272 | citrate | -0,0268 | -0,0497 |
| 273 | eicosapentaenoate (EPA; 20:5n3) | -0,0284 | 0,0095 |
| 274 | lysylvaline | -0,0299 | 0,0204 |
| 275 | 3'-dephosphocoenzyme A | -0,0305 | -0,0592 |
| 276 | nonadecanoate (19:0) | -0,0308 | 0,0297 |
| 277 | 5-dodecenoate (12:1n7) | -0,0317 | -0,0889 |
| 278 | fumarate | -0,033 | 0,0203 |
| 279 | oleic ethanolamide | -0,0335 | -0,0883 |
| 280 | glutathione, reduced (GSH) | -0,0357 | -0,0091 |
| 281 | N-stearoyl taurine | -0,0362 | -0,0259 |
| 282 | 5-methylthioadenosine (MTA) | -0,0383 | -0,0182 |
| 283 | methylphosphate | -0,0383 | 0,0916 |
| 284 | dihomo-linolenate (20:3n3 or n6) | -0,0384 | -0,0728 |
| 285 | 13-HODE + 9-HODE | -0,0389 | -0,0117 |
| 286 | threonylproline | -0,039 | -0,0028 |
| 287 | creatine | -0,0394 | -0,1051 |
| 288 | palmitoyl ethanolamide | -0,0396 | -0,0799 |
| 289 | adrenate (22:4n6) | -0,0416 | -0,0081 |
| 290 | N6-methyladenosine | -0,0442 | 0,0712 |
| 291 | citrulline | -0,0453 | 0,05 |
| 292 | adenosine | -0,0459 | 0,0347 |
| 293 | myristate (14:0) | -0,0483 | -0,0474 |
| 294 | 1-stearoylglycerophosphoinositol | -0,0496 | 0,0084 |
| 295 | cholesterol | -0,0497 | 0,0424 |
| 296 | stearoyl sphingomyelin | -0,0519 | 0,0256 |
| 297 | 3-dehydrocarnitine* | -0,0526 | -0,0784 |
| 298 | sedoheptulose-7-phosphate | -0,0553 | 0,0264 |
| 299 | xylulose | -0,0565 | 0,0319 |
| 300 | succinylcarnitine | -0,0582 | -0,0619 |
| 301 | cytidine-3'-monophosphate (3'-CMP) | -0,0589 | 0,0005 |
| 302 | carnitine | -0,0593 | -0,0979 |
| 303 | nicotinamide | -0,0593 | -0,0003 |
| 304 | docosapentaenoate (n6 DPA; 22:5n6) | -0,0595 | -0,0205 |
| 305 | eicosenoate (20:1n9 or 11) | -0,0602 | 0,0407 |
| 306 | ribulose | -0,0614 | 0,0038 |
| 307 | phosphopantetheine | -0,0618 | -0,0481 |
| 308 | 2-methylmalonyl carnitine | -0,0632 | -0,0761 |
| 309 | adenosine 5'-diphosphate (ADP) | -0,0651 | -0,0443 |
| 310 | carnosine | -0,0665 | 0,048 |
| 311 | 1-stearoylglycerophosphoethanolamine | -0,0666 | 0,0306 |
| 312 | histidine | -0,0669 | 0,0338 |
| 313 | adenine | -0,0693 | 0,0568 |
| 314 | glycerol | -0,0699 | -0,0214 |
| 315 | guanosine | -0,0702 | 0,0525 |
| 316 | mead acid (20:3n9) | -0,0708 | -0,0353 |
| 317 | malonylcarnitine | -0,0713 | -0,0631 |
| 318 | flavin mononucleotide (FMN) | -0,0728 | 0,0014 |
| 319 | linolenate [alpha or gamma; (18:3n3 or 6)] | -0,0728 | 0,02 |
| 320 | propionylcarnitine | -0,0729 | -0,0182 |
| 321 | uridine | -0,0733 | 0,0317 |
| 322 | S-adenosylhomocysteine (SAH) | -0,0749 | 0,0419 |
| 323 | hypoxanthine | -0,0763 | 0,0274 |
| 324 | N2-methylguanosine | -0,0772 | 0,0096 |
| 325 | inosine | -0,078 | 0,024 |
