# Supplementary material for: Global Metabolomic Profiling of Acute Myocarditis Caused by Trypanosoma cruzi Infection
Source: PLoS Negl Trop Dis. 2014 Nov 20;8(11):e3337. doi: 10.1371/journal.pntd.0003337 (PMC4239010; doi:10.1371/journal.pntd.0003337)
Supplement: Table S3 — Contribution of the plasma individual biochemicals to the Principal components analysis (PCA). List of the calculated coefficients of heart tissue biochemicals ordered from higher to lower. The higher positive and negative coefficients are the ones that have more contribution for the PCA analysis. Component 1 may have the greatest contribution to separating the metabolic signature followed by component 2. Plasma candidate biomarkers as p-cresol sulphate, kynurenine and allantoin, which increase with the infection, with very high positive coefficients are highlighted. (DOCX) [file pntd.0003337.s003.docx]

**Table S3.**

|  | **Biochemical** | **Comp.1 [35.78%]** | **Comp.2 [15.40%]** |
| --- | --- | --- | --- |
| 1 | p-cresol sulfate | 0,0907 | 0,0134 |
| 2 | 2'-deoxycytidine | 0,0879 | -0,0405 |
| 3 | 2-hydroxy-3-methylvalerate | 0,083 | -0,0025 |
| 4 | corticosterone | 0,0821 | 0,0021 |
| 5 | alpha-hydroxyisovalerate | 0,0812 | -0,0064 |
| 6 | thymine | 0,0798 | -0,0316 |
| 7 | homocitrulline | 0,0793 | -0,044 |
| 8 | 1-methylhistidine | 0,0762 | -0,0359 |
| 9 | 4-methyl-2-oxopentanoate | 0,0762 | -0,058 |
| 10 | phenylacetylglycine | 0,0712 | -0,0271 |
| 11 | kynurenine | 0,0684 | -0,0789 |
| 12 | 7-alpha-hydroxy-3-oxo-4-cholestenoate (7-Hoca) | 0,0681 | 0,0102 |
| 13 | trans-urocanate | 0,066 | 0,0141 |
| 14 | 1-methylimidazoleacetate | 0,0624 | 0,0216 |
| 15 | N-acetyl-beta-alanine | 0,0623 | -0,0293 |
| 16 | 2-hydroxyisobutyrate | 0,0583 | -0,0521 |
| 17 | thymidine | 0,0562 | -0,0796 |
| 18 | phenyllactate (PLA) | 0,056 | -0,044 |
| 19 | allantoin | 0,0557 | -0,0518 |
| 20 | propionylcarnitine | 0,0499 | -0,034 |
| 21 | fucose | 0,047 | -0,0551 |
| 22 | phenol sulfate | 0,0468 | -0,0963 |
| 23 | 3-methyl-2-oxovalerate | 0,0431 | -0,0606 |
| 24 | 3-hydroxyisobutyrate | 0,0427 | -0,0883 |
| 25 | isobutyrylcarnitine | 0,0427 | -0,084 |
| 26 | N-acetylalanine | 0,042 | -0,0501 |
| 27 | C-glycosyltryptophan* | 0,0397 | -0,0646 |
| 28 | 7-methylguanine | 0,0394 | -0,0746 |
| 29 | pseudouridine | 0,038 | -0,01 |
| 30 | creatinine | 0,0352 | 0,0532 |
| 31 | indolelactate | 0,0348 | -0,1009 |
| 32 | cytidine | 0,0346 | -0,0786 |
| 33 | uracil | 0,0344 | -0,0551 |
| 34 | 3-methyl-2-oxobutyrate | 0,0327 | -0,0849 |
| 35 | dimethylarginine (SDMA + ADMA) | 0,0321 | -0,0918 |
| 36 | urea | 0,0303 | -0,0251 |
| 37 | valine | 0,0303 | -0,0734 |
| 38 | tauro(alpha + beta)muricholate | 0,0287 | 0,0004 |
| 39 | kynurenate | 0,0272 | -0,0196 |
| 40 | 2-hydroxybutyrate (AHB) | 0,0271 | -0,0332 |
| 41 | pantothenate | 0,0263 | -0,1056 |
| 42 | carnitine | 0,0259 | 0,044 |
| 43 | N1-methyladenosine | 0,0166 | -0,0419 |
| 44 | alpha-muricholate | 0,0163 | 0,0146 |
| 45 | taurocholate | 0,0121 | -0,0069 |
| 46 | sarcosine (N-Methylglycine) | 0,0113 | -0,0446 |
| 47 | 2'-deoxyuridine | 0,011 | -0,0872 |
| 48 | adenosine | 0,0094 | 0,0037 |
| 49 | taurohyodeoxycholic acid | 0,0084 | 0,0634 |
| 50 | ornithine | 0,0067 | -0,0793 |
| 51 | benzoate | 0,0066 | 0,0775 |
| 52 | 4-hydroxyphenylpyruvate | 0,0064 | -0,1171 |
| 53 | isoleucine | 0,0055 | -0,0744 |
| 54 | deoxycholate | 0,0045 | 0,0529 |
| 55 | 4-hydroxymandelate | 0,0042 | -0,0981 |
| 56 | 3-(4-hydroxyphenyl)lactate | 0,004 | -0,0844 |
| 57 | hexadecanedioate | 0,0032 | 0,075 |
| 58 | caprylate (8:0) | 0,0024 | 0,0615 |
| 59 | octadecanedioate | 0,0024 | 0,0803 |
| 60 | 3-ureidopropionate | 0,001 | -0,0769 |
| 61 | phosphate | 0,0007 | 0,1325 |
| 62 | cholate | 0,0004 | 0,0336 |
| 63 | 3-hydroxybutyrate (BHBA) | -0,0029 | -0,0436 |
| 64 | heptanoate (7:0) | -0,0045 | 0,1065 |
| 65 | arabinose | -0,0048 | -0,0035 |
| 66 | 2-methylbutyrylcarnitine (C5) | -0,0064 | -0,0595 |
| 67 | urate | -0,0088 | -0,109 |
| 68 | taurodeoxycholate | -0,0092 | 0,0067 |
| 69 | anserine | -0,0118 | -0,0243 |
| 70 | 2-aminoheptanoic acid | -0,0119 | 0,0576 |
| 71 | myristoleate (14:1n5) | -0,013 | 0,0624 |
| 72 | caproate (6:0) | -0,0135 | 0,1074 |
| 73 | palmitoylcarnitine | -0,0144 | -0,09 |
| 74 | caprate (10:0) | -0,015 | 0,0606 |
| 75 | pelargonate (9:0) | -0,0155 | 0,1165 |
| 76 | 1-dihomo-linoleoylglycerophosphocholine (20:2n6)* | -0,0159 | -0,0037 |
| 77 | 4-hydroxy-2-nonenal | -0,0159 | 0,0804 |
| 78 | threonate | -0,0163 | -0,0137 |
| 79 | docosadienoate (22:2n6) | -0,0168 | 0,0099 |
| 80 | creatine | -0,0184 | -0,074 |
| 81 | isovalerylcarnitine | -0,0191 | -0,0506 |
| 82 | valerylcarnitine | -0,02 | 0,0082 |
| 83 | pipecolate | -0,0201 | -0,1286 |
| 84 | 3-indoxyl sulfate | -0,0205 | -0,0866 |
| 85 | adrenate (22:4n6) | -0,0207 | 0,0165 |
| 86 | myo-inositol | -0,0207 | -0,0687 |
| 87 | pyruvate | -0,021 | 0,0005 |
| 88 | tetradecanedioate | -0,0215 | 0,1187 |
| 89 | choline | -0,0222 | -0,0239 |
| 90 | hydroxyisovaleroyl carnitine | -0,0229 | -0,033 |
| 91 | dimethylglycine | -0,0232 | -0,0701 |
| 92 | leucine | -0,0247 | -0,0995 |
| 93 | N6-acetyllysine | -0,0247 | 0,0303 |
| 94 | 3-hydroxyoctanoate | -0,025 | 0,0982 |
| 95 | 10-undecenoate (11:1n1) | -0,0273 | -0,0058 |
| 96 | N-acetylphenylalanine | -0,0276 | -0,0343 |
| 97 | N1-methylguanosine | -0,0279 | -0,0087 |
| 98 | 2-linoleoylglycerol (2-monolinolein) | -0,0281 | 0,0617 |
| 99 | pyridoxate | -0,0305 | 0,0022 |
| 100 | xylose | -0,0307 | 0,0572 |
| 101 | gamma-glutamylalanine | -0,0311 | -0,0772 |
| 102 | hydroxybutyrylcarnitine* | -0,0312 | -0,0194 |
| 103 | benzyl alcohol | -0,0321 | -0,0616 |
| 104 | glycerate | -0,0325 | 0,0738 |
| 105 | N-acetylisoleucine | -0,0329 | 0,0049 |
| 106 | 7-beta-hydroxycholesterol | -0,0331 | 0,086 |
| 107 | acetylcarnitine | -0,0333 | -0,0485 |
| 108 | indoleacrylate | -0,0339 | -0,1109 |
| 109 | ethyl glucuronide | -0,0346 | 0,0049 |
| 110 | phenylalanine | -0,0346 | -0,0833 |
| 111 | 1-palmitoleoylglycerophosphocholine (16:1)* | -0,0347 | -0,0959 |
| 112 | xanthosine | -0,0351 | 0,0729 |
| 113 | 2-stearoylglycerophosphocholine* | -0,0358 | -0,0904 |
| 114 | 7-alpha-hydroxycholesterol | -0,0358 | 0,0803 |
| 115 | pinitol | -0,0362 | -0,0628 |
| 116 | 5-methylcytidine | -0,0373 | -0,0012 |
| 117 | 1-palmitoylglycerophosphocholine (16:0) | -0,038 | -0,1124 |
| 118 | 1-docosahexaenoylglycerophosphocholine (22:6n3)* | -0,0384 | -0,1039 |
| 119 | 13-HODE + 9-HODE | -0,0385 | 0,1256 |
| 120 | palmitoleate (16:1n7) | -0,0394 | 0,0491 |
| 121 | N-acetylglycine | -0,0398 | -0,005 |
| 122 | raffinose | -0,0403 | -0,046 |
| 123 | 12,13-hydroxyoctadec-9(Z)-enoate | -0,0404 | 0,0741 |
| 124 | 1-stearoylglycerophosphocholine (18:0) | -0,0408 | -0,097 |
| 125 | 1-margaroylglycerophosphocholine (17:0) | -0,041 | -0,0947 |
| 126 | 1-oleoylglycerophosphocholine (18:1) | -0,0416 | -0,1113 |
| 127 | 5-methylthioadenosine (MTA) | -0,0425 | -0,0082 |
| 128 | salicylate | -0,0426 | 0,0179 |
| 129 | gamma-glutamylphenylalanine | -0,0429 | -0,0997 |
| 130 | ribitol | -0,0431 | -0,0466 |
| 131 | 2-aminoadipate | -0,0433 | -0,082 |
| 132 | N-delta-acetylornithine* | -0,0434 | -0,0527 |
| 133 | 1-eicosatrienoylglycerophosphocholine (20:3)* | -0,0436 | -0,1054 |
| 134 | laurate (12:0) | -0,0437 | 0,0509 |
| 135 | 2-hydroxydecanoic acid | -0,0443 | 0,0586 |
| 136 | gamma-glutamylmethionine | -0,0455 | -0,0924 |
| 137 | 4-hydroxyhippurate | -0,0459 | -0,1032 |
| 138 | 2-aminooctanoate | -0,0464 | -0,032 |
| 139 | 3-phenylpropionate (hydrocinnamate) | -0,0466 | -0,0068 |
| 140 | phenylpropionylglycine | -0,0469 | -0,0862 |
| 141 | 2-piperidinone | -0,0471 | 0,0477 |
| 142 | 2-(4-hydroxyphenyl)propionate | -0,0474 | -0,1064 |
| 143 | gamma-glutamylvaline | -0,0475 | -0,0347 |
| 144 | serotonin (5HT) | -0,0476 | -0,0589 |
| 145 | gamma-glutamylleucine | -0,0477 | -0,088 |
| 146 | 1-linolenoylglycerophosphocholine (18:3n3)* | -0,0485 | -0,0781 |
| 147 | myristate (14:0) | -0,0488 | 0,0718 |
| 148 | docosahexaenoate (DHA; 22:6n3) | -0,049 | 0,0044 |
| 149 | gamma-glutamylisoleucine* | -0,049 | -0,0573 |
| 150 | erythronate* | -0,0492 | -0,077 |
| 151 | 3-hydroxypropanoate | -0,0499 | -0,0022 |
| 152 | TDTEDKGEFLSEGGGVR* | -0,0503 | 0,0452 |
| 153 | 2-linoleoylglycerophosphocholine* | -0,0505 | -0,0886 |
| 154 | N-acetylthreonine | -0,0506 | -0,0027 |
| 155 | glutamine | -0,0515 | -0,0213 |
| 156 | dihomo-linoleate (20:2n6) | -0,0517 | 0,0398 |
| 157 | 4-hydroxyphenylacetate | -0,0518 | -0,0656 |
| 158 | tryptophan | -0,0519 | -0,096 |
| 159 | 3-phosphoglycerate | -0,052 | 0,092 |
| 160 | linolenate [alpha or gamma; (18:3n3 or 6)] | -0,052 | 0,0609 |
| 161 | flavin adenine dinucleotide (FAD) | -0,0525 | 0,0066 |
| 162 | 2-ethylhexanoate | -0,0527 | -0,088 |
| 163 | gamma-glutamylglutamine | -0,053 | -0,0776 |
| 164 | 1-arachidonoylglycerophosphocholine (20:4n6)* | -0,0535 | -0,0896 |
| 165 | azelate (nonanedioate) | -0,0544 | -0,0155 |
| 166 | 1-linoleoylglycerophosphocholine (18:2n6) | -0,0546 | -0,0945 |
| 167 | N-acetylleucine | -0,0548 | -0,0509 |
| 168 | 12-HETE | -0,055 | 0,0935 |
| 169 | 1-stearoylglycerophosphoethanolamine | -0,055 | -0,1024 |
| 170 | erythritol | -0,055 | -0,0362 |
| 171 | oleate (18:1n9) | -0,055 | 0,0885 |
| 172 | 1-palmitoylglycerophosphoethanolamine | -0,0551 | -0,1059 |
| 173 | 5-oxoproline | -0,0559 | 0,0057 |
| 174 | docosapentaenoate (n3 DPA; 22:5n3) | -0,0566 | 0,0009 |
| 175 | gulono-1,4-lactone | -0,0566 | -0,015 |
| 176 | tyrosine | -0,0567 | -0,0844 |
| 177 | mannitol | -0,0571 | 0,0464 |
| 178 | pentadecanoate (15:0) | -0,0571 | 0,0933 |
| 179 | 3-hydroxyhippurate | -0,0573 | -0,001 |
| 180 | S-adenosylhomocysteine (SAH) | -0,0574 | 0,0561 |
| 181 | linoleate (18:2n6) | -0,0575 | 0,0671 |
| 182 | inosine | -0,0576 | 0,06 |
| 183 | 2-palmitoylglycerophosphoethanolamine* | -0,0579 | -0,0946 |
| 184 | gamma-glutamyltyrosine | -0,0582 | -0,0886 |
| 185 | sebacate (decanedioate) | -0,0582 | -0,0034 |
| 186 | 2-oleoylglycerophosphocholine* | -0,0583 | -0,077 |
| 187 | N-acetylglutamine | -0,0588 | -0,0186 |
| 188 | pyrophosphate (PPi) | -0,059 | -0,0191 |
| 189 | 1-linoleoylglycerol (1-monolinolein) | -0,0596 | 0,0521 |
| 190 | 1,3-dipalmitoylglycerol | -0,0603 | 0,0611 |
| 191 | catechol sulfate | -0,0606 | -0,0507 |
| 192 | fructose | -0,0613 | -0,0156 |
| 193 | N2-acetyllysine | -0,0613 | 0,0638 |
| 194 | 1,2-dipalmitoylglycerol | -0,0615 | 0,0469 |
| 195 | 2-aminophenol sulfate | -0,0617 | -0,0426 |
| 196 | butyrylcarnitine | -0,062 | -0,0484 |
| 197 | eicosenoate (20:1n9 or 11) | -0,062 | 0,0384 |
| 198 | proline | -0,0627 | -0,0546 |
| 199 | glutamate | -0,0634 | 0,0136 |
| 200 | inositol 1-phosphate (I1P) | -0,064 | -0,0038 |
| 201 | hexanoylcarnitine | -0,0642 | -0,0128 |
| 202 | homostachydrine* | -0,0642 | 0,0178 |
| 203 | asparagine | -0,0643 | -0,0155 |
| 204 | N-acetylaspartate (NAA) | -0,0643 | 0,033 |
| 205 | hexanoylglycine | -0,0645 | -0,027 |
| 206 | mannose | -0,0645 | 0,0305 |
| 207 | nicotinamide | -0,0647 | 0,0187 |
| 208 | arabitol | -0,0649 | -0,0039 |
| 209 | histidine | -0,0651 | 0,0409 |
| 210 | stearidonate (18:4n3) | -0,0652 | 0,0542 |
| 211 | 10-heptadecenoate (17:1n7) | -0,0653 | 0,0662 |
| 212 | 2-arachidonoylglycerophosphoethanolamine* | -0,0656 | -0,0561 |
| 213 | homoserine | -0,0656 | -0,0793 |
| 214 | pro-hydroxy-pro | -0,0656 | 0,0292 |
| 215 | lysine | -0,0657 | -0,0854 |
| 216 | 4-guanidinobutanoate | -0,0663 | 0,0283 |
| 217 | xylonate | -0,0663 | 0,0281 |
| 218 | hippurate | -0,0665 | -0,0377 |
| 219 | glutathione, oxidized (GSSG) | -0,0667 | 0,0365 |
| 220 | N-acetyltyrosine | -0,0667 | -0,0455 |
| 221 | methionine | -0,067 | -0,0752 |
| 222 | 2-oxindole-3-acetate | -0,0678 | -0,0546 |
| 223 | 4-hydroxybutyrate (GHB) | -0,0681 | 0,0549 |
| 224 | equol glucuronide | -0,0682 | -0,0138 |
| 225 | 2-aminobutyrate | -0,0683 | -0,0281 |
| 226 | palmitate, methyl ester | -0,0686 | -0,0528 |
| 227 | beta-hydroxypyruvate | -0,0689 | 0,0231 |
| 228 | valerylglycine | -0,0689 | 0,0217 |
| 229 | palmitoyl sphingomyelin | -0,0694 | -0,0052 |
| 230 | 1-oleoylglycerophosphoethanolamine | -0,0699 | -0,0789 |
| 231 | cis-vaccenate (18:1n7) | -0,0699 | 0,0486 |
| 232 | S-methylglutathione | -0,0703 | 0,0197 |
| 233 | docosapentaenoate (n6 DPA; 22:5n6) | -0,0704 | 0,04 |
| 234 | N-acetyltryptophan | -0,0708 | 0,0013 |
| 235 | trans-4-hydroxyproline | -0,0709 | -0,0028 |
| 236 | carnosine | -0,0719 | 0,0238 |
| 237 | methylphosphate | -0,0719 | 0,0634 |
| 238 | succinate | -0,0719 | 0,0292 |
| 239 | N-acetylhistidine | -0,0726 | -0,0237 |
| 240 | 10-nonadecenoate (19:1n9) | -0,0728 | 0,0604 |
| 241 | 17-methylstearate | -0,0728 | 0,0178 |
| 242 | 1-palmitoylglycerophosphoinositol* | -0,0729 | 0,021 |
| 243 | eicosapentaenoate (EPA; 20:5n3) | -0,0729 | 0,0073 |
| 244 | cysteine-glutathione disulfide | -0,073 | 0,0523 |
| 245 | dihomo-linolenate (20:3n3 or n6) | -0,0733 | 0,0089 |
| 246 | N-formylmethionine | -0,0738 | 0,0242 |
| 247 | 1-linoleoylglycerophosphoethanolamine* | -0,0739 | -0,0497 |
| 248 | 1-stearoylglycerophosphoinositol | -0,0744 | 0,0584 |
| 249 | butyrylglycine | -0,0745 | 0,0213 |
| 250 | cinnamoylglycine | -0,0745 | -0,0285 |
| 251 | campesterol | -0,0747 | 0,0431 |
| 252 | glycerol | -0,0748 | -0,0006 |
| 253 | uridine | -0,0748 | 0,0295 |
| 254 | 1-arachidonoylglycerophosphoethanolamine* | -0,0749 | -0,0535 |
| 255 | betaine | -0,0749 | 0,0113 |
| 256 | glycerol 2-phosphate | -0,0749 | 0,0312 |
| 257 | isovalerylglycine | -0,075 | 0,029 |
| 258 | alanylalanine | -0,0753 | -0,0095 |
| 259 | 2-linoleoylglycerophosphoethanolamine* | -0,0754 | -0,0471 |
| 260 | indolepropionate | -0,0755 | -0,0675 |
| 261 | glycine | -0,0757 | -0,049 |
| 262 | threonine | -0,0757 | -0,0469 |
| 263 | ribose | -0,0759 | -0,0003 |
| 264 | TDTEDKGEFLSEGGGV* | -0,0762 | 0,0041 |
| 265 | 2-oleoylglycerophosphoethanolamine* | -0,0765 | -0,0497 |
| 266 | mead acid (20:3n9) | -0,0766 | 0,0248 |
| 267 | alanine | -0,0772 | -0,0531 |
| 268 | alpha-ketoglutarate | -0,0774 | 0,0248 |
| 269 | lactate | -0,0774 | -0,0181 |
| 270 | 3-dehydrocarnitine* | -0,0775 | 0,0229 |
| 271 | 1-palmitoylplasmenylethanolamine* | -0,0779 | -0,0318 |
| 272 | stearoyl sphingomyelin | -0,0781 | -0,0011 |
| 273 | margarate (17:0) | -0,0783 | 0,0501 |
| 274 | 2-hydroxyglutarate | -0,0784 | 0,0067 |
| 275 | nonadecanoate (19:0) | -0,0787 | 0,0303 |
| 276 | stachydrine | -0,0797 | -0,015 |
| 277 | citrulline | -0,0805 | -0,0545 |
| 278 | 2-hydroxypalmitate | -0,081 | 0,0022 |
| 279 | arginine | -0,0815 | -0,0187 |
| 280 | xylitol | -0,0815 | 0,0099 |
| 281 | gluconate | -0,0816 | -0,0309 |
| 282 | 1-linoleoylglycerophosphoinositol* | -0,082 | 0,047 |
| 283 | sorbitol | -0,0827 | 0,0348 |
| 284 | malate | -0,0831 | 0,0014 |
| 285 | beta-alanine | -0,0836 | -0,0175 |
| 286 | arachidonate (20:4n6) | -0,0839 | 0,0082 |
| 287 | ribulose | -0,084 | -0,0042 |
| 288 | 2-hydroxystearate | -0,0843 | -0,0272 |
| 289 | trigonelline (N'-methylnicotinate) | -0,0843 | -0,0121 |
| 290 | equol sulfate | -0,0847 | -0,0012 |
| 291 | citrate | -0,0852 | -0,006 |
| 292 | N-acetylmethionine | -0,0855 | -0,0174 |
| 293 | palmitate (16:0) | -0,0861 | 0,046 |
| 294 | 1-arachidonoylglycerophosphoinositol* | -0,0863 | 0,0338 |
| 295 | fumarate | -0,0863 | 0,0084 |
| 296 | deoxycarnitine | -0,0866 | -0,0089 |
| 297 | cholesterol | -0,087 | 0,0225 |
| 298 | stearate (18:0) | -0,0871 | 0,0389 |
| 299 | cis-aconitate | -0,0872 | -0,0088 |
| 300 | serine | -0,0874 | -0,0129 |
| 301 | 1,5-anhydroglucitol (1,5-AG) | -0,0877 | 0,0041 |
| 302 | glucose | -0,0888 | 0,0167 |
| 303 | glycerophosphorylcholine (GPC) | -0,0888 | 0,0265 |
| 304 | glycerol 3-phosphate (G3P) | -0,0892 | 0,0041 |
| 305 | glycolate (hydroxyacetate) | -0,0895 | 0,0109 |
| 306 | aspartate | -0,0905 | 0,0149 |
